# The Microbiota and Evolution of Obesity

**DOI:** 10.1210/endrev/bnae033

**Published:** 2024-12-13

**Authors:** Mario J A Saad, Andrey Santos

**Affiliations:** Department of Internal Medicine, School of Medical Sciences, University of Campinas, CEP 13083-887 Campinas, SP, Brazil; Department of Internal Medicine, School of Medical Sciences, University of Campinas, CEP 13083-887 Campinas, SP, Brazil

**Keywords:** gut microbiota, obesity, evolution, metaflammation, drift and thrifty

## Abstract

Obesity is a major global concern and is generally attributed to a combination of genetic and environmental factors. Several hypotheses have been proposed to explain the evolutionary origins of obesity epidemic, including thrifty and drifty genotypes, and changes in thermogenesis. Here, we put forward the hypothesis of metaflammation, which proposes that due to intense selection pressures exerted by environmental pathogens, specific genes that help develop a robust defense mechanism against infectious diseases have had evolutionary advantages and that this may contribute to obesity in modern times due to connections between the immune and energy storage systems. Indeed, incorporating the genetic variations of gut microbiota into the complex genetic framework of obesity makes it more polygenic than previously believed. Thus, uncovering the evolutionary origins of obesity requires a multifaceted approach that considers the complexity of human history, the unique genetic makeup of different populations, and the influence of gut microbiome on host genetics.

## Graphical Abstract

**Graphical Abstract:**
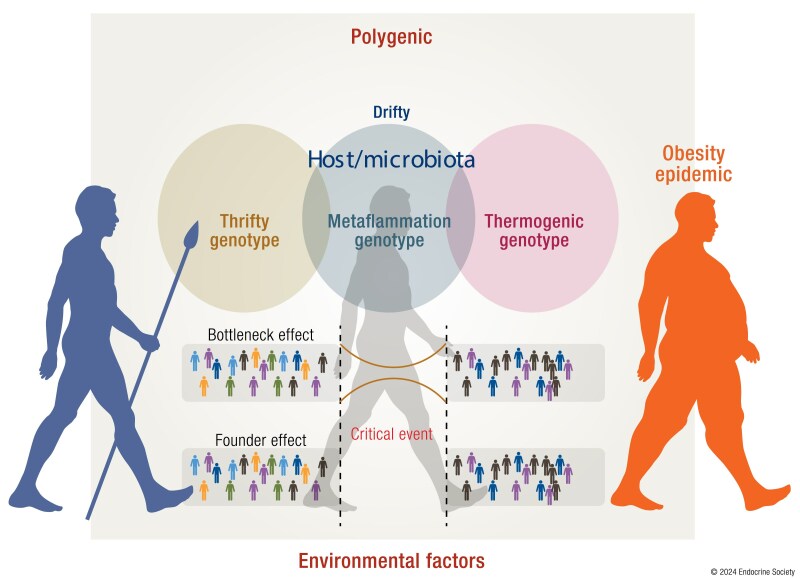

**Essential Points**

- Various hypotheses have been suggested for the evolutionary origins of obesity
- Metaflammation suggests that pathogen defense genes could lead to modern obesity
- Immune genes may influence obesity via immune and energy storage system interactions
- Gut microbiota genetics add to obesity's polygenic complexity
- Obesity's origins require considering history, genetics, and the gut microbiota

Over the past 4 decades, the obesity epidemic has rapidly escalated in Western societies. While obesity clearly has many environmental drivers including the changing nature of diets, it also has a substantial genetic component; tracing the evolutionary origins of this genetic history remains an important challenge (1-3).

Charles Darwin and Alfred Wallace revolutionized the understanding of how different environmental exposures in previous generations shaped biological diversity in today's generation. Their theory of natural selection proposed that species evolve, giving rise to new species, while sharing a common ancestry (4), and that this evolution is driven by natural selection, which favors traits that enhance the fitness of the species (5-7). However, it was not until the late 19th century that modern genetics emerged with the rediscovery of Mendel's work, laying the foundation for modern evolutionary synthesis.

Throughout history, humans have faced ever-changing environmental and social conditions, both before and after their migration out of Africa. Factors such as predation, famine, infectious diseases, and climate adaptation have shaped human evolution. However, with the rapid changes in lifestyle in recent years, the levels of daily activity and type/quality of food intake have become maladaptive. Applying these evolutionary concepts to explain the modern epidemics of obesity and type 2 diabetes have traditionally focused on genetic traits. With the completion of the Human Genome Project, our understanding of the genetic traits has advanced considerably (8). Indeed, over the last 15 years, researchers investigating the genetics of obesity using large populations and the genome-wide association studies (GWAS) approach have identified more than 1000 genetic loci linked to obesity (9). Despite such advancements, the exact driver genes of the most common types of obesity and their mechanism of action are not yet fully understood (9). It is possible that integrating these modern genetic studies with the hypothesis of the evolutionary origins of obesity can be one path to shed light on the role of genetics in obesity.

In this review, we revisit the existing hypotheses that, to some extent, explain the evolutionary basis of recent obesity epidemics, including the thrifty and drifty genotypes (1, 2) and the thermogenic hypothesis (3). Due to the intense selection pressures caused by environmental pathogens, we have put forward an additional metaflammation hypothesis in which modern-day obesity may also be driven in part from natural selection to favor specific genes that promote strong immune defense against epidemics and/or infectious diseases in our ancestors. Considering the close connection between the immune and energy storage systems (10), these genes might allow for efficient fat storage during food-abundance periods, allowing more resilience in times of stress. In today's constant food availability environment, however, this inflammatory genotype or metaflammation promotes excessive fat storage and obesity. However, it is essential to emphasize that the origins of obesity are complex and cannot be explained by a single theory.

Thus far, the effect of the interplay between host and microbial genetic variation on host evolution has received little attention in the study of obesity. Most of the research on obesity has largely neglected the microbiome's influence on the genetic basis and evolution of the host. Here we propose investigating how genetic variations in the microbiome can increase the genetic diversity of the host genome, affect the heritability of host traits, and ultimately influence the evolution of obesity in humans. Integrating data from the GWAS and the microbiome into these previous hypotheses, we explore the need to consider the changing nature of microbiota in the critical process of evolution that converges to our modern epidemic of obesity.

## Evolutionary Hypothesis of Obesity

### Thrifty Genotype

The oldest hypothesis, proposed by Neel in 1962, suggests that diabetes and obesity may have originated from natural selection to favor a “thrifty genotype” in our ancestors (1). This genotype would allow for efficient fat storage during food-abundance periods, which was advantageous when surviving food shortages. However, in today's constant food availability environment, this thrifty genotype would promote excessive fat storage and obesity (11-22).

One of the many criticisms of the hypothesis is that the causes of mortality are complex during times of famine, with significant factors being infectious disease and diarrhea (23), suggesting that mechanisms of immune defense against infection need to be included in the search for genotypes with evolutionary advantages associated with the thrifty genotype. Further weakening the thrifty genotype is the dearth of genetic studies supporting this hypothesis (24-26). Moreover, Wang and Speakman (27), who searched for genetic evidence of the thrifty genotype in the positive selection signatures at 115 single-nucleotide polymorphisms (SNPs) linked to obesity found no selection evidence, and thereby no support for the thrifty genotype as a major evolutionary driver for obesity.

### Drifty Hypothesis

The drifty hypothesis, proposed by Speakman, challenges the concept of the thrifty genotype to explain obesity (2). Based on this hypothesis, it is suggested that early hominids underwent a process of stabilizing selection favoring body fatness, while obesity was selected against due to the increased risk of predation. However, around 2 million years ago, the risk of predation diminished substantially with the development of social behavior, weapons, and fire control. As a result, the population distribution of body fatness began to alter due to random mutations and genetic drift (2, 28, 29).

In essence, the drifty hypothesis suggests that once our ancestors became skilled hunters and discovered fire, the risk of predation reduced and was nearly nonexistent. This removal of predation as a selection pressure meant that the upper limit or “point of intervention” for body weight status was no longer beneficial. Thus, the genes that promote adiposity and increased body weight were no longer being removed by natural selection, as they had been when predation posed a severe threat to survival. This hypothesis differs notably from the previous one by suggesting that the genetic predisposition to obesity has never been advantageous to humans (11, 16, 20, 28-32).

The hypothesis also explains why most individuals in society are not obese. Potential genetic alterations that cause upper body weight limits to be exceeded are presumed to have randomly occurred rather than being selected for. Therefore, individuals who have not experienced this genetic drift remain nonobese (2). Critics of this hypothesis argue that it fails to consider factors such as population size, gene-gene and gene-environment interactions, population bottlenecks and expansions, migration and founder effects, and population subdivision (33). Additionally, the hypothesis does not address certain genetic traits, such as type 2 diabetes and polycystic ovary syndrome, which are highly detrimental in our environment and cannot be solely explained by random mutations (34).

### Thermogenic Capacity Hypothesis

Compelling evidence now suggests that modern humans embarked on a remarkable journey out of Africa approximately 70 000 years ago (35-45). As our ancestors ventured into colder regions (Europe and Northeast Asia), they faced unique environmental challenges that shaped their genetic makeup. Over time, natural selection favored genes that facilitated cold adaptation over heat adaptation (3, 46).

It is worth noting that modern humans reached Europe around 45 000 years ago and inhabited it at a time of the last glacial period when vast stretches of Europe were engulfed by ice. Around 40 000 years ago, Europe experienced a climatic deterioration that reduced mammalian species diversity. Ethnographic data and observations on mammalian species and fluctuating resources indicate a subsequent decline in human population densities, and suggest that population bottlenecks, genetic drift, and gene flow have more prominent roles in human evolution during this period than population replacement.

As a result, populations that remained in Africa were well adapted to hot climates and local savannah environments—features found even in modern times in individuals of African descent, including a larger surface area to body mass ratio, longer limbs, increased skin pigmentation, reduced body hair, more sweat glands, lower body temperature, and decreased metabolic rate, all of which would have helped protect individuals against solar radiation and overheating (47, 48). In contrast, indigenous populations with ancestors from China and Japan successfully settled in Arctic and subarctic regions, showcasing their evolutionary adaptation to cold climates (49-51). It is believed that natural selection has played a role in favoring cold-adaptation genes in these populations, influencing energy expenditure in these individuals with diverse ancestries (3). These studies have found that basal metabolic rates are highest in Arctic individuals, intermediate in White Europeans, and lowest in African Americans (52-54). These findings underpin a thermogenic capacity hypothesis (55-61), which suggests that the lineages of early humans who remained in Africa and those who migrated to other tropical environments retained heat-adaptation genes (3). As a result, modern African Americans, whose ancestors did not require such efficient energy expenditure, showed lower aerobic capacity and energy expenditure, which, when combined with sedentary Western lifestyles, increased obesity rates (52, 62). Indeed, total daily energy expenditure is lower in African American compared with White individuals, most of which is due to a lower resting metabolic rate (52, 62). Conversely, the lineages of those who migrated to colder regions acquired genes for cold adaptation (3, 63). Despite sedentary lifestyles and ultraprocessed foods, populations adapted to cold temperatures and with a propensity to efficient energy expenditure have less chance of developing obesity when compared with populations in hot climates.

Thus, the thermogenic capacity hypothesis highlights the profound influence of historical human migration on the modern obesity pandemic. The journey of our ancestors out of Africa, coupled with unique climatic challenges, has shaped distinct genetic adaptations in different populations. In accordance with this hypothesis, there are some gene variants associated with latitude, obesity, and brown adipose tissue thermogenesis, such as *UCP1*, *PRDM16*, *THADA*, *ADRB3*, *TBX15/Wars2*, and *TRIB2* (58). While the hypothesis provides valuable insights into human evolution and its effect on metabolic rates and obesity, as noted later, further research is needed to determine how differences and changes in gut microbiota might contribute to these differences and reinforce the hypothesis.

### A New Hypothesis: The Metaflammation Hypothesis

Due to intense selection pressures exerted by pathogens, the immune system has become our primary interface with the environment (64-66). Devastating historical epidemics, such as the Black Death in Europe, viruses that decimated Native Americans in Peru and Mexico, and the influenza pandemic of 1919, have had a significant effect on population sizes and genetic selection (66). Disparities in obesity rates exist among different populations, with African Americans, Hispanic Americans, and Pacific Islanders having higher rates when compared to European Americans (67). Together, these observations lead us to propose a metaflammation hypothesis, which proposes that obesity rate differences between populations reside, at least in part, in the differences in the immune system (which is linked to the energy storage system) and are the consequences of genetic selection induced by infectious diseases or epidemics. Thus, populations that stayed in Africa and lived a more primal lifestyle, hunting in tropical rainforests where they were exposed to various parasites and pathogens carried by insects, birds, and animals, have developed a robust immune system (68, 69). By contrast, populations that migrated out of Africa were exposed to lower pathogen levels, thereby reducing the need for strong and energy-costly proinflammatory signals (70, 71).

In favor of this hypothesis, it has been shown that individuals of African descent, including African Americans, express more genes linked to strong inflammation, increased cytokine secretion, and bactericidal activities when compared to other populations (65, 72). There are more than 250 such genes with evidence of recent natural selection, for example, variants of the *IL1A* and *IL1B* genes (65, 72). Macrophages are required to fight infections and in individuals of African ancestry, macrophages respond more strongly to infections, as assessed by expression of genes related to inflammatory responses (65). These findings suggest that Africans and African Americans have more efficient inflammatory responses and may better control bacterial infections.

Recent studies have shown that Hispanic Americans, who have a high prevalence of obesity, have inherited stronger immune systems from their Native American ancestors, possibly because the latter had survived epidemics of infectious disease. Research has also shown that African American and Hispanic American women have higher circulating C-reactive protein levels when compared to European American women. This phenomenon is linked to a specific protein variant (TREM2) which is expressed in myeloid cells (73).

Another population with a high obesity prevalence, which likely experienced selective pathogen pressure, are the Pima Indians. GWAS studies conducted in this population have identified multiple SNPs associated with body mass index (BMI), including SNPs in *A2BP1*, *TMEM18*, *TCF7L2*, *MAP2K3*, and *LPGAT1* (74-77). Although these genes had many different cellular functions, most of these are expressed in macrophages and/or code for proteins that can modulate the immune responses or are related to endoplasmic reticulum stress (76, 78-80). Thus, these genes could have roles in subclinical inflammation in obesity and also serve as connections between inflammatory genotypes and weight gain.

In the 19th century, infectious diseases such as measles, whooping cough, and influenza caused approximately 75% mortality in some East Polynesian populations (81), and potentially exerted a considerable effect on genetic diversity in modern populations. In GWAS of obese populations from the Pacific Islands, strong associations were observed with *Insig2* and *CREBRF* genes (82, 83), which, while not uniquely related to the immune system, have relevant roles in inflammation or endoplasmic reticulum stress directly linked to inflammatory responses (84, 85).

Although less prevalent than in African Americans and Hispanic Americans, Europeans and European Americans also have a high prevalence of obesity. While most GWAS in obese populations of European ancestry have not reported correlations between BMI and immune system genes, a more careful search can identify possible links. For example, two of the most significant GWAS-identified and widely replicated obesity loci are the *FTO* (9, 86) and *MC4R* genes (9, 87, 88). Although several mechanisms have been proposed to explain why these loci modulate body weight, including the central nervous system–mediated control of food intake (9), it is important to note that both *FTO* (89-98) and *MC4R* (99-102) have important roles in macrophage activation and inflammatory responses, suggesting some effect on immune response modulation. Moreover, reexamination of a study examining the genetic factors contributing to BMI variations in 339 000 individuals (103) (predominantly of European descent) using GWAS and metabochip meta-analysis to successfully identify 97 BMI-associated loci, which accounted for approximately 2.7% of the variance in BMI, revealed many expected pathways, including substantial central nervous system involvement, but also revealed 56 novel loci associated with BMI in a European meta-analysis, of which at least 90% had roles in macrophage/inflammatory processes, indicating potential connections between BMI and immune genotype composition (104-137).

While it is commonly believed that subclinical inflammation is caused by obesity in response to cytokines secreted from macrophage/adipose tissue, epidemiological studies have shown that inflammation can precede and promote weight gain (138-141). At molecular levels, precise control mechanisms exist between insulin signaling/resistance and pathways in immune cells that may contribute to weight gain. In primary infections or excess nutrient conditions, innate immune system activation (toll-like receptor [TLR], inducible nitric oxide synthase, JNK, and nuclear factor κB) causes posttranscriptional protein modifications in insulin signaling. This causes insulin resistance, which is specific to the liver, muscle, and hypothalamus, while adipose tissue remains insulin sensitive or less resistant thereby favoring weight gain (141-144). Inflammation may also contribute to increased weight gain via reduced energy expenditure, secondary to M1 macrophage infiltration in brown adipose tissue, thereby increasing degradation or impairing sympathetic neuron-mediated norepinephrine signaling in this tissue (145, 146).

In summary, our metaflammation hypothesis suggesting that genes that promote a strong defense against infectious diseases could also be responsible for the increasing prevalence of obesity in modern society. This hypothesis could also shed light on the evolutionary origins of the obesity epidemic, but further studies of the connections between genes linked to obesity and the immune system and inflammation must be explored.

## Microbiota, Obesity, and Evolution

### Environment Factors and Microbiota

The environment is a determinant factor in the establishment of the obesity pandemic observed in recent years. Nevertheless, the increase in obesity cannot be entirely ascribed to individual choices for high-calorie diets or decreased energy expenditure resulting from contemporary sedentary lifestyles. This viewpoint overemphasizes personal responsibility for obesity, failing to acknowledge the broader systemic factors that contribute to the creation of inequitable obesogenic environments. For instance, the unique characteristics of Latin American countries render their populations particularly susceptible to these factors, which may elucidate the substantial increase in obesity rates observed in the region (147-149). These factors include the physical environment, food exposure, economic and political interests, social inequity, limited access to scientific knowledge, cultural influences, contextual behavior, and genetics (147, 150-153). While some factors are related to individual behavior, most are systemic, significantly affecting obesity trends by limiting individual freedom of choice. Additionally, the reduced selective pressure resulting from medical advancements and food abundance may allow individuals with a genetic predisposition to obesity to survive and reproduce, potentially increasing obesity prevalence in future generations (154).

**Figure 1.**
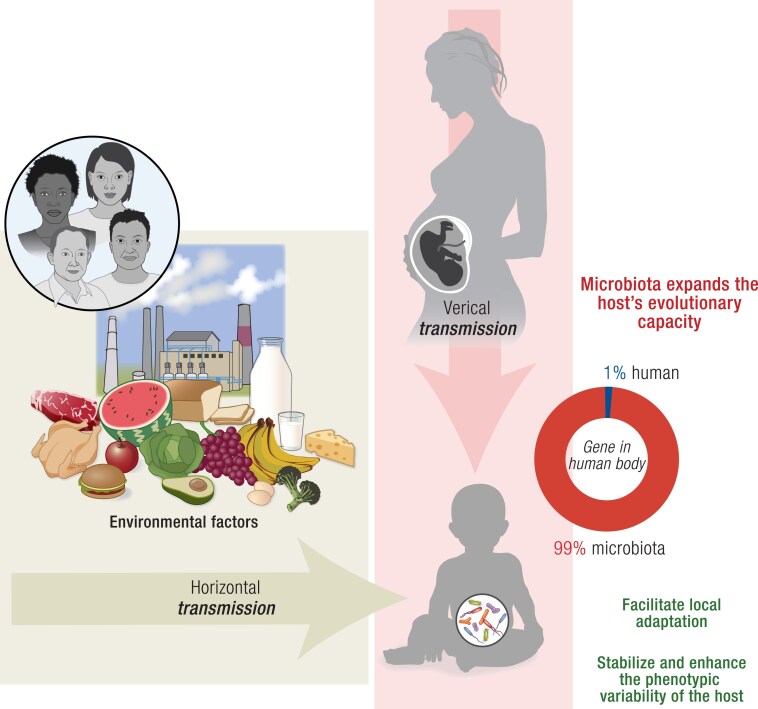
The gut microbiome expands the host’s evolutionary capacity. The study of human evolution is incomplete without considering the human microbiota. Bacterial cells in gut microbiota possess 99% of the genetic information in our bodies, providing us with augmented genetic diversity compared to our genome and facilitating accelerated evolutionary processes through generations. Previously, only vertically transmitted microbes were recognized as inheritable, but hosts can also acquire gut microbiota through horizontal gene transfer. The microbiome can modulate the host's evolutionary potential in 2 common scenarios: First, microbial variation may shift the population's mean phenotype, facilitating local adaptation, and second, microbial diversity can stabilize and enhance phenotypic variability within a host population. The effect of the microbiome on host genetics needs to be considered in the hypotheses of the evolutionary origin of obesity.

Also in this field of evolutionary biology, recent evidence suggests that the study of human evolution is incomplete without due consideration of the human microbiota (155-159). The gut microbiota is a complex ecosystem of gastrointestinal microorganisms, including bacteria, viruses, fungi, protozoa, and archaea. More than a trillion microorganisms, including normal commensal bacteria in various compartment of the body, influence the functioning of the human body (160-162). Of these, gut bacteria have been studied the most. In addition to maintaining normal intestinal function, intestinal microbiota also influences the overall health of the host (160-169). Bacterial cells from the gut microbiota possess an astounding number of genes that surpasses the entire human genome (160, 162, 170-172). As a result, they have gained the moniker “second genome” or “extended genotype.” This secondary genetic system can account for an overwhelming 99% of the genetic information in our bodies, providing us with augmented genetic diversity compared to our genome (Fig. 1). Moreover, it facilitates accelerated evolutionary processes and grants us the remarkable capability of exchanging microorganisms with our surroundings, along with their genes and associated functionalities (173, 174). These attributes hold immense potential in contributing to the adaptability of the host organism, making the second genome an appealing target for natural selection.

Diet and lifestyle choices substantially influence the gut microbiota (175-183) and have profound implications on the evolutionary journey toward obesity. Vertebrates, including humans, modulate their intestinal microbiota in response to acute and chronic dietary changes (175, 177, 178, 184, 185). This adaptation enables greater flexibility and efficiency in digesting a wide range of nutrients, promoting survival even under extreme dietary conditions. Evidence indicates the existence of diurnal oscillations in the gut microbiota of mice and humans, corresponding to feeding rhythms (185-187), as well as long-term adaptations that provide a mechanism for responding to changing environments and providing evolutionary influence on the host. This is exemplified by comparison of gut microbiota between populations from the United States, Malawi, and the Amazon and their adaptation to differing dietary components (188). People in America have adapted to a high-protein and high-fat diet, whereas individuals from Malawi and the Amazon have adapted to digest complex carbohydrates.

Horizontal gene transfer represents another adaptation of the human microbiota with substantial implications in evolution (see Fig. 1). This transfer involves changes in the composition of bacteria within the gut and subsequent alterations in gene content (189). Furthermore, it has been demonstrated that human-associated bacteria have a substantially higher rate of gene transfer than bacteria in other environments, because horizontal gene transfer occurs frequently within an individual's gut microbiome, with higher frequencies of transfer in industrialized populations (190).

In addition to diet, various environmental factors, including early-life antibiotic use, treatment with antipsychotic medications, smoking cessation, reduced physical activity, and numerous other conditions, have been shown to affect the composition of gut microbiota, potentially favoring weight gain (191-207). Host genetic variation also contributes to shape the microbial ecosystem (208-214). This interaction between host genetics and the gut microbiome can potentially affect the host's phenotype. Understanding the complex interplay between human genetics, environment, and gut microbiota provides valuable insights into the evolutionary origins of obesity and its underlying mechanisms.

### Gut Microbiota and Obesity

Extensive research has shed light on the critical role of intestinal microbiota in the development of obesity (162, 215-219). In a now classic study, it was found that germ-free mice were comparatively protected against diet-induced obesity and exhibited reduced adiposity, improved glucose tolerance, and enhanced insulin sensitivity, all linking the microbiome to obesity and metabolic syndrome (217). Transplantation of the microbiota from ob/ob mice to lean mice increased adiposity in the recipients, even though they did not carry the obesity genes (219). Indeed, multiple studies in different mice models suggest the causal role of microbiota as an important variable in the induction of weight gain (220, 221), and indicate that intestinal microbiota may overcome genetic protection against insulin resistance, inducing weight gain and metabolic syndrome (222).

In humans, the composition and biodiversity of gut bacteria substantially differ between obese and healthy individuals (196, 223-241). Compared to lean individuals, obese individuals show reduced bacterial diversity. A systematic review showed that the most consistent phylum associated with obesity is Proteobacteria, and the association between the Bacteroidetes/Firmicutes ratio is dubious (241). In obesity, various genera, such as Lactobacillus and Fusobacterium, are also enriched. On the other hand, Faecalibacterium, Akkermansia, and Alistipes are considered to be lean-associated genera (242-244). In a metagenome-wide association study, researchers have found 1358 significant associations between bacterial SNPs and host body mass index (BMI) using gut metagenomic samples from a cohort of more than 7000 healthy individuals (245). The researchers also identified BMI associations in SNPs related to inflammatory pathways in Bilophila wadsworthia and energy metabolism functions in the *Faecalibacterium prausnitzii* genome, highlighting the significance of nucleotide-level diversity in microbiome studies.

### Gut Microbiome Expands the Host’s Evolutionary Capacity

The microbiome plays an important role in the host's evolutionary potential by expanding its genetic repertoire (156-158, 246-248). The interaction of the microbiome with the host phenotype is crucial in shaping the distribution of host phenotypes. It enhances the host's response to natural selection and influences its evolutionary trajectory. Microbial effects on host evolution depend on how microbes are transferred to the host species. Previously, only vertically transmitted microbes were recognized as inheritable; however, hosts can acquire microbes through different transmission modes (249) (see Fig. 1). Recent research has revealed that host genetic variation significantly contributes to the relative abundance of microbes in hosts that acquire microbiome directly from the environment (209, 250, 251). On the other side, despite the complex inheritance of the microbiome, microbial variation explains considerable phenotypic variance that can rival the contribution of host genetic, suggesting that the microbiome's fidelity of inheritance may also influence host phenotypic variance (208, 209).

The microbiome can modulate the host's evolutionary potential in two common scenarios (252). First, microbial variation may shift the mean phenotype of the population, facilitating local adaptation (173, 252, 253). Second, microbial diversity has the potential to stabilize and enhance phenotypic variability within a host population. These two patterns often coexist and significantly influence how hosts navigate their adaptive journey (253, 254). By harnessing the abilities of microbes, hosts can acquire specific adaptive traits tailored to their local environment, thereby maximizing their chances of survival and reproductive success in rapidly changing ecological landscapes (156).

The assembly process of intestinal microbiota introduces chance and priority effects, resulting in microbial variation among hosts within a population. Therefore, it increases phenotypic variability and creates new opportunities for host exploration within the fitness landscape. This alteration in evolutionary trajectories has important implications for hosts. Thus, changes in the distribution of phenotypic traits within the microbiome affect the host's response to natural selection, leading to tractable signatures of selection in the host's genome over time. The interplay between microbial variation and host phenotypic diversity plays a crucial role in the dynamics of evolutionary processes. These findings highlight the critical role of the microbiome in shaping the adaptive potential of host populations and provide valuable insights into the intricate interdependencies between microbes and their hosts.

While locally adaptive microbes may help facilitate short-term host trait evolution, their long-term evolutionary outcomes are still unknown. If these microbes prove beneficial, hosts may develop mechanisms to maintain locally adaptive microbes and their effects on host traits or environmental stress mitigation, similar to genetic accommodation or niche construction (253). Thus, hosts may increase their frequency within the population, improving the host's ability to adapt to the environment.

Specifically looking at the immune system, protective symbionts potentially shape immune system evolution in multiple ways. One possibility is that host immune responses, when coupled with protective symbionts, reduce the need for redundant immune mechanisms. In contrast, symbionts may also help develop host immune responses by providing sufficient protection, thereby enabling hosts to persist and adapt. Immune system evolution is likely to differ, depending on factors such as the type of immunity, how symbionts are transmitted, and the cost benefits associated with immune system functions. Ultimately, the effect of beneficial symbiosis on immunity evolution will rely on the intricate interactions between the host immune system and symbionts, with specific interactions potentially alleviating the pressure for immune system maintenance, while others may create constraints (255).

## Reconciling the Evolutionary Hypothesis of Obesity With the Changing Landscape of Gut Microbiota

### Host/Microbiota Thrifty Hypothesis

**Figure 2.**
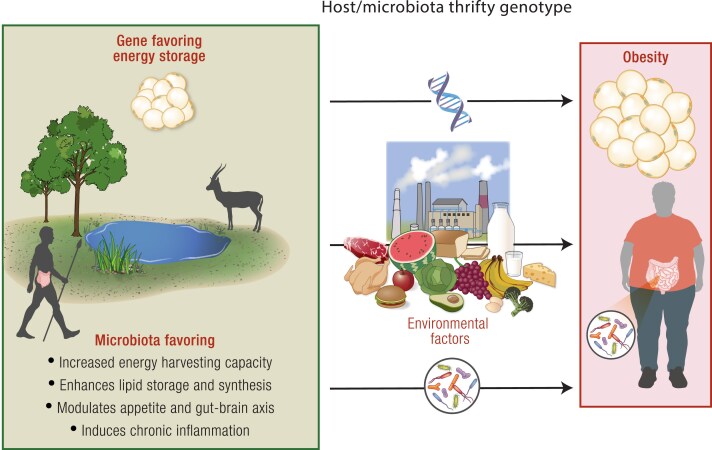
Host/microbiota thrifty genotype hypothesis. The host thrifty genotype proposes that genes that allowed for efficient fat storage during food abundance were advantageous for survival during periods of food shortage. Additionally, a microbiota able to induce energy storage and less energy expenditure, independent of whether it was installed more recently (favored by environmental factors) or was installed in our ancestors and passed through vertical transmission, can undoubtedly integrate the thrifty genotype. A possible microbiota thrifty genotype is a microbiota favoring a) an increase in digestible energy uptake while decreasing energy expenditure, leading to weight gain; b) an increase in lipid synthesis and storage, contributing to obesity; c) the control of appetite and feeding behavior and modulate the gut-brain axis to influence cravings and eating habits; d) the induction of a state of subclinical chronic inflammation, leading to tissue-specific insulin resistance with increased adipose mass. In today's environment of constant food availability, this host/microbiota thrifty genotype promotes excessive fat storage and obesity.

The discovery of the important and changing role of gut microbiota in the development of obesity provides a new mechanism that must be considered in the context of the thrifty genotype hypothesis. Mechanisms, whereby gut microbiota can promote weight gain/energy storage, can be categorized into 4 key areas. First, gut microbiota disrupt energy homeostasis by increasing digestible energy uptake (increased capacity to energy harvest) (219), leading to weight gain. Second, gut microbiota may enhance lipid synthesis and storage, contributing to obesity (217). Furthermore, gut microbiota may affect control of appetite and feeding behavior and modulate the gut-brain axis to influence cravings and eating habits (256, 257). Last, gut microbiota may induce a state of subclinical chronic inflammation, leading to tissue-specific insulin resistance with increased adipose mass (141, 258-261). A gut microbiota with these characteristics is deemed to have a “thrifty genotype,” which refers to its ability to efficiently induce fat storage in the host (141, 217, 219, 256-261), a trait that have been advantageous in our ancestors (Fig. 2).

### Host/Microbiota Thermogenic Capacity Hypothesis

Previous data have shown that cold exposure can lead to a substantial shift in mouse microbiota composition, which researchers dubbed the “cold microbiota” (262). Intriguingly, when these microbiota were transplanted into germ-free mice, the animals showed improved insulin sensitivity and better cold tolerance effects that were partly due to white fat browning and increased energy expenditure with loss of white fat. Ziętak et al (263) found that lowering the environmental temperature reduced diet-induced obesity in mice and was associated with increased thermogenesis and a plasma bile acid profile similar to their germ-free counterparts. The authors observed significant changes in microbiome composition at both the phylum and family levels within a day of cold exposure and after 4 weeks at lower temperatures. Interestingly, under these conditions, the gut microbiota showed higher levels of bacteria associated with leanness, such as *Adlercreutzia*, *Mogibacteriaceae*, *Ruminococcaceae*, and *Desulfovibrio*, while bacteria linked to obesity (*Bacilli*, *Erysipelotrichaceae*, and rc4-4) were reduced.

**Figure 3.**
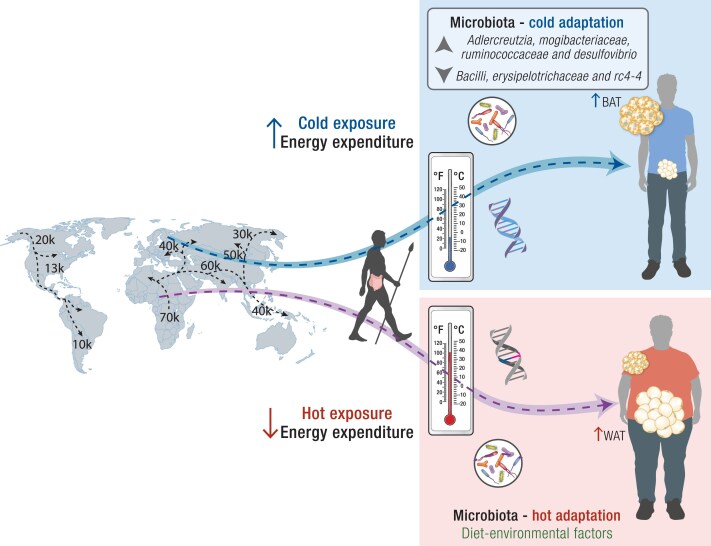
Host/microbiota thermogenic genotype hypothesis: The thermogenic capacity hypothesis suggests that the lineage of early humans who remained in Africa and those who migrated to other tropical environments retained genes for heat adaptation. Conversely, the lineage of those who migrated to colder regions acquired genes for cold adaptation. Nowadays, with a sedentary lifestyle and ultraprocessed food abundance, populations adapted to cold temperatures with a propensity to efficient energy expenditure have less chance to develop obesity compared with populations that were adapted to hot climates. In addition, it is essential to mention that exposure to cold temperatures induces alterations in the microbiota composition that favor genera associated with leanness and suppresses those linked to obesity. This leads to the hypothesis that the modulation of microbiota could potentially explain the adaptation of White and East Asian individuals to cold climates and their resistance to obesity. Furthermore, the opposite modulation of microbiota in hot climates may predispose descendants (coupled with sedentary and Western lifestyles) to obesity. This phenomenon can be identified as the “host/microbiota thermogenic capacity genotype” adaptation, which may also elucidate the relatively rapid development of obesity when these populations migrate from cold to hot climates, accompanied by lifestyle changes.

Taken together, these findings suggest that exposure to cold temperatures induce microbiota composition alterations that favor genera associated with leanness and suppress those linked to obesity (262, 264, 265). Thus, changes in microbiota can potentially explain, at least in part, White European and East Asian adaptation to cold climates and their resistance to obesity. Furthermore, in hot climates, microbiota modulation in the opposite direction, coupled with sedentary and Western lifestyles, may contribute to an obesity propensity among African and South Asian populations. This “host/microbiota thermogenic capacity genotype” adaptation may also contribute to relatively rapid obesity development when these populations migrate from cold to hot climates (Fig. 3). Such lifestyle changes may represent a promising avenue for further research in this field.

### Host/Microbiota Metaflammation Hypothesis

The host and its commensal bacteria work together to resist pathogens, with cooperative efforts potentially favored by natural selection (266-269). Pathogen defenses are crucial microbiome functions in terms of evolution, and many symbionts that have colonized hosts are effective against a range of pathogens, making the benefits of pathogen resistance a considerable advantage (255, 270). This is particularly important compared to other microbiota benefits, such as nutritive benefits or the thrifty microbiota genotype.

In this regard, a careful search using data from different sources shows that intestinal microbiota taxa considered protective against some infectious diseases are more prevalent in microbiota from obese individuals (240, 271, 272). There is a clear relationship between the gut microbiota and the sepsis outcome (273-275). A mendelian randomization investigation estimates that *Lentisphaerae*, *LachnospiraceaeUCG004*, and *Coprococcus* negatively correlated with sepsis severity. In addition, *Coprococcus* had a significant negative correlation with the risk of sepsis-related death, suggesting a protective effect of these taxa (271). Interestingly, all these taxa are more prevalent in obese individuals, suggesting that, at least in part, a more protective microbiota in sepsis is also present in obesity (147). A systematic review of malaria and microbiome showed a clear correlation between the phylum firmicutes and proteobacteria and the attenuation of malaria severity in mice and men (272), and these phyla are certainly more prevalent also in obesity (240). Although the microbiota of obese individuals might have a significant influence from diet and environment, we cannot exclude the possibility that part of it may have come from vertical transmission, which leads us to suggest that certain microbiota strains that have evolutionary advantages in fighting infectious diseases may also predispose the host to weight gain.

**Figure 4.**
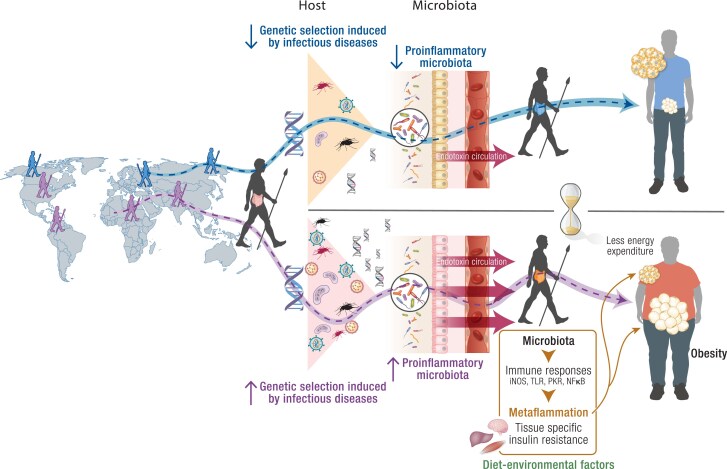
Host/microbiota metaflammation genotype hypothesis: There are disparities in obesity rates among different populations, with African Americans, Hispanic Americans, and Pacific Islanders having higher rates compared to European Americans. The differences in obesity prevalence between human populations may involve the immune response, which lies in the genetic selection induced by infectious diseases. As populations stayed in Africa, they lived and hunted in the tropical rainforest. They were exposed to various insect, bird, and animal parasites and pathogens and developed a more inflammatory genotype. As some humans migrated out of Africa to develop agriculture and animal husbandry, they encountered diverse pathogenic environments. This led to population-specific selection and adaptation to these new environments, with less pressure on infectious diseases and a less inflammatory genotype. Evidence shows that a genotype more prone to inflammation may predispose to obesity in today's constant food availability environment. Moreover, we suggest that certain strains of microbiota that possess evolutionary advantages in fighting infectious diseases may contribute to a more inflammatory phenotype, predisposing to weight gain, reinforcing the role of a more inflammatory microbiota in the evolutionary origins of obesity (microbiota metaflammation genotype). Taken together, we propose the integration of host and microbiota genotypes and call it the host/microbiota metaflammation genotype hypothesis.

The colonization resistance induced by gut microbiota may involve direct mechanisms (interactions between microbial cells) and indirect mechanisms (through regulation of host physiology and largely host immune responses) (276). These indirect mechanisms, mainly activation of the innate immune system and cytokine production, may also mediate weight gain. Individuals with low gut bacterial diversity have low-grade inflammation due to innate immune system activation and are more likely to experience weight gain, dyslipidemia, and insulin resistance (277, 278). Also, specific bacterial strains associated with host inflammation, such as *Ruminococcus gnavus* and *Bacteroides* species, are more prevalent in obese individuals (178, 279). In contrast, strains with anti-inflammatory properties (*F prausnitzii*) are less common (192). Furthermore, a unique intestinal microbiome signature was shown to contribute to weight regain in obese mice following successful dieting (280). The molecular connections between microbiota-induced inflammation and obesity may be manifested through factors previously described, such as tissue-specific insulin resistance and reduced energy expenditure (141-146, 281-283), but other microbiota-related mechanisms are also likely at play. One additional potential mechanism involves fatty acid metabolism by the gut microbiota and its effect on the obesity-inflammation axis. Research has shown that dietary and microbial factors influence specific fatty acid isomer levels in the gut, which modulate specific immune cells called CD4+ intraepithelial lymphocytes (284). These findings provide a new role for bacterial fatty acid metabolism in maintaining the immunological balance in the gut by modulating the relative number of CD4+ T cells that are CD4+ CD8αα+. These studies support the notion that distinct gut microbial signatures are associated with host inflammation and obesity. Taken together with these data, we can suggest that the microbiota exhibiting these characteristics can be identified as possessing a “metaflammation genotype,” which is responsible for its ability to combat infections and promote fat storage in the host effectively (Fig. 4).

### COVID-19 Pandemic and the Metaflamation Hypothesis

The recent COVID-19 pandemic needs to be analyzed considering this new metaflammation hypothesis. First, it is important to mention that the pandemic of the 21st century is very different from those of previous centuries, considering the availability of vaccines and medical and hospital resources, including intensive care, which are much more advanced today. However, some data from the COVID-19 pandemic seemingly support the metaflammation hypothesis. To begin with, the recent pandemic induced an acute pronounced inflammatory response in patients followed in some of them by a milder chronic inflammatory process, which has been termed “*long COVID*.” In these patients, weight gain was observed in the months following the initial episode (285-289), confirming that a nonsevere but chronic inflammatory process can lead to weight gain through the mechanisms previously described (138-146). As expected, GWAS studies conducted in this population have identified multiple SNPs associated mainly with the immune system (290-295), again indicating the connection between the immune response and the energy storage system (adipose tissue).

Additionally, it is important to highlight that patients experiencing long COVID exhibit gut microbiota dysbiosis, characterized by a significant reduction in bacterial diversity. This includes a lower relative abundance of genera known to confer protection against obesity, particularly those that produce short-chain fatty acids, such as the *Eubacterium hallii* group, *Subdoligranulum*, *Ruminococcus*, *Dorea*, *Coprococcus*, and the *Eubacterium ventriosum* group (296, 297). On the other side, the relative abundance of *Veillonella*, which is a genus abundant in individuals with a high inflammatory index (298), was higher compared to controls. A recent study (299) used summary statistics from GWAS and mendelian randomization analyses, aiming to explore the association between gut microbiota and long COVID. The meta-analysis findings indicated that the genus *Parasutterella* significantly elevated the risk of developing long COVID. In this context, previous research has demonstrated a positive correlation between *Parasutterella* and both BMI and type 2 diabetes, independent of the reduced microbiome alpha and beta diversity and the low-grade inflammation typically observed in obesity (300). Taking together these data, we can suggest that the immune response to an infection is a complex process that involves the genetic architecture of the immune system and the microbiota, and epidemics may select survivors with a more robust inflammatory response that can predispose to obesity even in future generations.

In summary, we are suggesting that the evolutionary hypotheses of obesity should be enriched with microbiota genotype, and even for the drifty hypothesis (a nonadaptive scenario), microbiota modulation, mainly by environmental and dietetic factors more recently, certainly contributes to explaining the increased obesity prevalence in the past 40 years. Moreover, adding the microbiota genotype increases the scope of the thrifty, the thermogenic, and the metaflammation hypotheses in the adaptive scenario. However, it remains uncertain whether this microbiota genotype, or at least a portion of it, originated in the ancestors of obese individuals long ago and provided evolutionary benefits or if it is a more recent adaptation to our food-rich environment. Nonetheless, this microbial genotype found in obese individuals can be inherited by future generations, giving rise to a microbiota-associated thrifty, thermogenic, and metaflammation genotype.

## Conclusions

Human populations in different regions have unique genetic histories influenced by founder effects, genetic drift, admixture events, and various ecological challenges. These factors have collectively contributed to the genetic architecture of humans. It is crucial to acknowledge that models of the origin of obesity cannot be categorized as adaptive or nonadaptive. The origins of obesity are complex and cannot be explained by a single theory. Both natural selection and genetic drift likely influenced the genetic framework of obesity. There is an overlap of natural selection hypotheses that are not mutually exclusive. Natural selection may have increased the prevalence of beneficial alleles for survival, whereas genetic drift randomly affected the frequencies of other alleles. The combined effects of these forces and the modulation of microbiota under different circumstances may offer insight into the ethnogeographic variation in obesity. It is well accepted now that the common forms of obesity are polygenic, and incorporating the microbiota genotype in this complex genetic architecture certainly makes it more polygenic than previously thought. Thus, uncovering the evolutionary origins of obesity requires a multifaceted approach that considers the complexity of human history, the unique genetic makeup of different populations, and the influence of gut microbiome on host genetics. Exploring these factors together will open up new avenues for understanding the genetics of obesity and its evolution.

## Abbreviations

BMI: body mass index
GWAS: genome-wide association studies
SNP: single-nucleotide polymorphism

## References

[1] Neel JV . Diabetes mellitus: a “thrifty” genotype rendered detrimental by “progress”? Am J Hum Genet. 1962;14(4):353‐362. 13937884 PMC1932342

[2] Speakman JR . A nonadaptive scenario explaining the genetic predisposition to obesity: the “predation release” hypothesis. Cell Metab. 2007;6(1):5‐12. 17618852 10.1016/j.cmet.2007.06.004

[3] Sellayah D, Cagampang FR, Cox RD. On the evolutionary origins of obesity: a new hypothesis. Endocrinology. 2014;155(5):1573‐1588. 24605831 10.1210/en.2013-2103

[4] Darwin C . On the Origin of Species by Means of Natural Selection, or, the Preservation of Favoured Races in the Struggle for Life. John Murray; 1859. PMC5184128 30164232

[5] Dobzhansky T, Boesinger E. Human Culture: a Moment in Evolution. Columbia University Press; 1983.

[6] Dennett DC . Darwin's Dangerous Idea. Penguin; 1995.

[7] Hamilton WD . The genetical evolution of social behaviour. I. J Theor Biol. 1964;7(1):1‐16. 5875341 10.1016/0022-5193(64)90038-4

[8] Lander ES, Linton LM, Birren B, et al Initial sequencing and analysis of the human genome. Nature. 2001;409(6822):860‐921. 11237011 10.1038/35057062

[9] Loos RJF, Yeo GSH. The genetics of obesity: from discovery to biology. Nat Rev Genet. 2022;23(2):120‐133. 34556834 10.1038/s41576-021-00414-z PMC8459824

[10] Hotamisligil GS . Inflammation, metaflammation and immunometabolic disorders. Nature. 2017;542(7640):177‐185. 28179656 10.1038/nature21363

[11] Gupta MK, Gouda G, Vadde R. Relation between obesity and type 2 diabetes: evolutionary insights, perspectives and controversies. Curr Obes Rep. 2024;13(3):475‐495. 38850502 10.1007/s13679-024-00572-1

[12] Aisyah R, Sadewa AH, Patria SY, Wahab A. The PPARGC1A is the gene responsible for thrifty metabolism related metabolic diseases: a scoping review. Genes (Basel). 2022;13(10):1894. 36292779 10.3390/genes13101894 PMC9601628

[13] Johnson RJ, Sánchez-Lozada LG, Nakagawa T, et al Do thrifty genes exist? Revisiting uricase. Obesity (Silver Spring). 2022;30(10):1917‐1926. 36150210 10.1002/oby.23540 PMC9512363

[14] Garduño-Espinosa J, Ávila-Montiel D, Quezada-García AG, Merelo-Arias CA, Torres-Rodríguez V, Muñoz-Hernández O. Obesity and thrifty genotype. Biological and social determinism versus free will. Bol Med Hosp Infant Mex. 2019;76(3):106‐112. 31116710 10.24875/BMHIM.19000159

[15] Reddon H, Patel Y, Turcotte M, Pigeyre M, Meyre D. Revisiting the evolutionary origins of obesity: lazy versus peppy-thrifty genotype hypothesis. Obes Rev. 2018;19(11):1525‐1543. 30261552 10.1111/obr.12742

[16] Qasim A, Turcotte M, de Souza RJ, et al On the origin of obesity: identifying the biological, environmental and cultural drivers of genetic risk among human populations. Obes Rev. 2018;19(2):121‐149. 29144594 10.1111/obr.12625

[17] Reales G, Rovaris DL, Jacovas VC, et al A tale of agriculturalists and hunter-gatherers: exploring the thrifty genotype hypothesis in native South Americans. Am J Phys Anthropol. 2017;163(3):591‐601. 28464262 10.1002/ajpa.23233

[18] Vatsiou AI, Bazin E, Gaggiotti OE. Changes in selective pressures associated with human population expansion may explain metabolic and immune related pathways enriched for signatures of positive selection. BMC Genomics. 2016;17:504. 27444955 10.1186/s12864-016-2783-2 PMC4955149

[19] Myles S, Lea RA, Ohashi J, et al Testing the thrifty gene hypothesis: the Gly482Ser variant in PPARGC1A is associated with BMI in Tongans. BMC Med Genet. 2011;12:10. 21244673 10.1186/1471-2350-12-10 PMC3025936

[20] Speakman JR . Thrifty genes for obesity, an attractive but flawed idea, and an alternative perspective: the ‘drifty gene’ hypothesis. Int J Obes (Lond). 2008;32(11):1611‐1617. 18852699 10.1038/ijo.2008.161

[21] Carulli L, Rondinella S, Lombardini S, Canedi I, Loria P, Carulli N. Review article: diabetes, genetics and ethnicity. Aliment Pharmacol Ther. 2005;22(Suppl 2):16‐19. 16225465 10.1111/j.1365-2036.2005.02588.x

[22] Prentice AM, Rayco-Solon P, Moore SE. Insights from the developing world: thrifty genotypes and thrifty phenotypes. Proc Nutr Soc. 2005;64(2):153‐161. 15960860 10.1079/pns2005421

[23] Speakman JR . Thrifty genes for obesity and the metabolic syndrome–time to call off the search? Diab Vasc Dis Res. 2006;3(1):7‐11. 16784175 10.3132/dvdr.2006.010

[24] Minster RL, Hawley NL, Su CT, et al A thrifty variant in CREBRF strongly influences body mass index in Samoans. Nat Genet. 2016;48(9):1049‐1054. 27455349 10.1038/ng.3620 PMC5069069

[25] Southam L, Soranzo N, Montgomery SB, et al Is the thrifty genotype hypothesis supported by evidence based on confirmed type 2 diabetes- and obesity-susceptibility variants? Diabetologia. 2009;52(9):1846‐1851. 19526209 10.1007/s00125-009-1419-3 PMC2723682

[26] Wang L, Sinnott-Armstrong N, Wagschal A, et al A MicroRNA linking human positive selection and metabolic disorders. Cell. 2020;183(3):684‐701.e14. 33058756 10.1016/j.cell.2020.09.017 PMC8092355

[27] Wang G, Speakman JR. Analysis of positive selection at single nucleotide polymorphisms associated with body mass index does not support the “thrifty gene” hypothesis. Cell Metab. 2016;24(4):531‐541. 27667669 10.1016/j.cmet.2016.08.014

[28] Speakman JR . If body fatness is under physiological regulation, then how come we have an obesity epidemic? Physiology (Bethesda). 2014;29(2):88‐98. 24583765 10.1152/physiol.00053.2013

[29] Speakman JR . The evolution of body fatness: trading off disease and predation risk. J Exp Biol. 2018;221(Suppl 1):jeb167254. 29514887 10.1242/jeb.167254

[30] James WPT, Johnson RJ, Speakman JR, et al Nutrition and its role in human evolution. J Intern Med. 2019;285(5):533‐549. 30772945 10.1111/joim.12878

[31] Speakman JR, Hambly C. Using doubly-labelled water to measure free-living energy expenditure: some old things to remember and some new things to consider. Comp Biochem Physiol A Mol Integr Physiol. 2016;202:3‐9. 27040184 10.1016/j.cbpa.2016.03.017

[32] Speakman JR . Evolutionary perspectives on the obesity epidemic: adaptive, maladaptive, and neutral viewpoints. Annu Rev Nutr. 2013;33(1):289‐317. 23862645 10.1146/annurev-nutr-071811-150711

[33] Prentice AM, Hennig BJ, Fulford AJ. Evolutionary origins of the obesity epidemic: natural selection of thrifty genes or genetic drift following predation release? Int J Obes (Lond). 2008;32(11):1607‐1610. 18852700 10.1038/ijo.2008.147

[34] Diamond J . The double puzzle of diabetes. Nature. 2003;423(6940):599‐602. 12789325 10.1038/423599a

[35] Zhivotovsky LA, Rosenberg NA, Feldman MW. Features of evolution and expansion of modern humans, inferred from genomewide microsatellite markers. Am J Hum Genet. 2003;72(5):1171‐1186. 12690579 10.1086/375120 PMC1180270

[36] Armitage SJ, Jasim SA, Marks AE, Parker AG, Usik VI, Uerpmann HP. The southern route “out of Africa”: evidence for an early expansion of modern humans into Arabia. Science. 2011;331(6016):453‐456. 21273486 10.1126/science.1199113

[37] Ashraf Q, Galor O. The ‘out of Africa’ hypothesis, human genetic diversity, and comparative economic development. Am Econ Rev. 2013;103(1):1‐46. 25506083 10.1257/aer.103.1.1 PMC4262934

[38] Bons PD, Bauer CC, Bocherens H, et al Out of Africa by spontaneous migration waves. PLoS One. 2019;14(4):e0201998. 31013270 10.1371/journal.pone.0201998 PMC6478371

[39] Hershkovitz I, Weber GW, Quam R, et al The earliest modern humans outside Africa. Science. 2018;359(6374):456‐459. 29371468 10.1126/science.aap8369

[40] Bohár L, Nagy E, Buday P. [Possibilities of ultrasonic examination in traumatologic diagnosis]. Magy Traumatol Orthop Helyreallito Seb. 1985;28:42‐49. 2859395

[41] Hublin JJ, Ben-Ncer A, Bailey SE, et al New fossils from Jebel Irhoud, Morocco and the pan-African origin of homo sapiens. Nature. 2017;546(7657):289‐292. 28593953 10.1038/nature22336

[42] Hublin JJ . Recent human evolution in northwestern Africa. Philos Trans R Soc Lond B Biol Sci. 1992;337(1280):185‐191. 1357693 10.1098/rstb.1992.0096

[43] Richter D, Grün R, Joannes-Boyau R, et al The age of the hominin fossils from Jebel Irhoud, Morocco, and the origins of the middle stone age. Nature. 2017;546(7657):293‐296. 28593967 10.1038/nature22335

[44] Stringer C . Modern human origins: progress and prospects. Philos Trans R Soc Lond B Biol Sci. 2002;357(1420):563‐579. 12028792 10.1098/rstb.2001.1057 PMC1692961

[45] López S, van Dorp L, Hellenthal G. Human dispersal out of Africa: a lasting debate. Evol Bioinform Online. 2016;11(Suppl 2):57‐68. 27127403 10.4137/EBO.S33489 PMC4844272

[46] Hanna JM, Brown DE. Human heat tolerance: an anthropological perspective. Annu Rev Anthropol. 1983;12(1):259‐284.

[47] Thomson ML . A comparison between the number and distribution of functioning eccrine sweat glands in Europeans and Africans. J Physiol. 1954;123(2):225‐233. 13143505 10.1113/jphysiol.1954.sp005045 PMC1366196

[48] Moskowitz DW . Hypertension, thermotolerance, and the “African gene”: an hypothesis. Clin Exp Hypertens. 1996;18(1):1‐19. 8822230 10.3109/10641969609082603

[49] Takasaki Y, Loy SF, Juergens HW. Ethnic differences in the relationship between bioelectrical impedance and body size. J Physiol Anthropol Appl Human Sci. 2003;22(5):233‐235. 10.2114/jpa.22.233 14519912

[50] Snodgrass JJ, Sorensen MV, Tarskaia LA, Leonard WR. Adaptive dimensions of health research among indigenous Siberians. Am J Hum Biol. 2007;19(2):165‐180. 17286259 10.1002/ajhb.20624

[51] Milan FA, Evonuk E. Oxygen consumption and body temperatures of Eskimos during sleep. J Appl Physiol. 1967;22(3):565‐567. 6020243 10.1152/jappl.1967.22.3.565

[52] Sharp TA, Bell ML, Grunwald GK, et al Differences in resting metabolic rate between white and African-American young adults. Obes Res. 2002;10(8):726‐732. 12181380 10.1038/oby.2002.99

[53] Weyer C, Snitker S, Bogardus C, Ravussin E. Energy metabolism in African Americans: potential risk factors for obesity. Am J Clin Nutr. 1999;70(1):13‐20. 10393133 10.1093/ajcn/70.1.13

[54] Leonard WR, Sorensen MV, Galloway VA, et al Climatic influences on basal metabolic rates among circumpolar populations. Am J Hum Biol. 2002;14(5):609‐620. 12203815 10.1002/ajhb.10072

[55] Wu T, Xu S. Understanding the contemporary high obesity rate from an evolutionary genetic perspective. Hereditas. 2023;160(1):5. 36750916 10.1186/s41065-023-00268-x PMC9903520

[56] Hanson RL, Van Hout CV, Hsueh WC, et al Assessment of the potential role of natural selection in type 2 diabetes and related traits across human continental ancestry groups: comparison of phenotypic with genotypic divergence. Diabetologia. 2020;63(12):2616‐2627. 32886191 10.1007/s00125-020-05272-8 PMC7642101

[57] Salazar-Tortosa D, Fernández-Rhodes L. Obesity and climate adaptation. Evol Med Public Health. 2019;2019(1):104‐105. 31263561 10.1093/emph/eoz016 PMC6592263

[58] Sellayah D . The impact of early human migration on brown adipose tissue evolution and its relevance to the modern obesity pandemic. J Endocr Soc. 2019;3(2):372‐386. 30723844 10.1210/js.2018-00363 PMC6354082

[59] Nakayama K, Iwamoto S. An adaptive variant of TRIB2, rs1057001, is associated with higher expression levels of thermogenic genes in human subcutaneous and visceral adipose tissues. J Physiol Anthropol. 2017;36(1):16. 28212671 10.1186/s40101-017-0132-z PMC5316227

[60] Rotwein PS . Editorial: is it time for an evolutionarily based human endocrinology? Mol Endocrinol. 2015;29(4):487‐489. 25827340 10.1210/me.2015-1063 PMC4399278

[61] Albuquerque D, Stice E, Rodríguez-López R, Manco L, Nóbrega C. Current review of genetics of human obesity: from molecular mechanisms to an evolutionary perspective. Mol Genet Genomics. 2015;290(4):1191‐1221. 25749980 10.1007/s00438-015-1015-9

[62] Carpenter WH, Fonong T, Toth MJ, et al Total daily energy expenditure in free-living older African-Americans and Caucasians. Am J Physiol. 1998;274(1):E96‐E101. 9458753 10.1152/ajpendo.1998.274.1.E96

[63] Hallmark B, Karafet TM, Hsieh P, Osipova LP, Watkins JC, Hammer MF. Genomic evidence of local adaptation to climate and diet in indigenous siberians. Mol Biol Evol. 2019;36(2):315‐327. 30428071 10.1093/molbev/msy211

[64] Barreiro LB, Quintana-Murci L. From evolutionary genetics to human immunology: how selection shapes host defence genes. Nat Rev Genet. 2010;11(1):17‐30. 19953080 10.1038/nrg2698

[65] Nédélec Y, Sanz J, Baharian G, et al Genetic ancestry and natural selection drive population differences in immune responses to pathogens. Cell. 2016;167(3):657‐669.e21. 27768889 10.1016/j.cell.2016.09.025

[66] Karlsson EK, Kwiatkowski DP, Sabeti PC. Natural selection and infectious disease in human populations. Nat Rev Genet. 2014;15(6):379‐393. 24776769 10.1038/nrg3734 PMC4912034

[67] Centers for Disease Control and Prevention, N. C. f. C. D. P. a. H. P . Division of Population Health. BRFSS Prevalence & Trends Data [online]. 2015. Accessed February 21, 2024. https://www.cdc.gov/brfss/brfssprevalence/

[68] Mercer A . Protection against severe infectious disease in the past. Pathog Glob Health. 2021;115(3):151‐167. 33573529 10.1080/20477724.2021.1878443 PMC8079125

[69] McNeill WH . Plagues and Peoples. Penguin Books; 1979.

[70] Guernier V, Hochberg ME, Guégan JF. Ecology drives the worldwide distribution of human diseases. PLoS Biol. 2004;2(6):e141. 15208708 10.1371/journal.pbio.0020141 PMC423130

[71] Barreiro LB, Ben-Ali M, Quach H, et al Evolutionary dynamics of human toll-like receptors and their different contributions to host defense. PLoS Genet. 2009;5(7):e1000562. 19609346 10.1371/journal.pgen.1000562 PMC2702086

[72] Ness RB, Haggerty CL, Harger G, Ferrell R. Differential distribution of allelic variants in cytokine genes among African Americans and white Americans. Am J Epidemiol. 2004;160(11):1033‐1038. 15561982 10.1093/aje/kwh325

[73] Reiner AP, Beleza S, Franceschini N, et al Genome-wide association and population genetic analysis of C-reactive protein in African American and hispanic American women. Am J Hum Genet. 2012;91(3):502‐512. 22939635 10.1016/j.ajhg.2012.07.023 PMC3511984

[74] Ma L, Hanson RL, Traurig MT, et al Evaluation of A2BP1 as an obesity gene. Diabetes. 2010;59(11):2837‐2845. 20724578 10.2337/db09-1604 PMC2963542

[75] Muller YL, Hanson RL, Piaggi P, et al Assessing the role of 98 established Loci for BMI in American Indians. Obesity (Silver Spring). 2019;27(5):845‐854. 30887699 10.1002/oby.22433 PMC6478540

[76] Bian L, Traurig M, Hanson RL, et al MAP2K3 is associated with body mass index in American Indians and Caucasians and may mediate hypothalamic inflammation. Hum Mol Genet. 2013;22(21):4438‐4449. 23825110 10.1093/hmg/ddt291 PMC3792696

[77] Traurig MT, Orczewska JI, Ortiz DJ, et al Evidence for a role of LPGAT1 in influencing BMI and percent body fat in native Americans. Obesity (Silver Spring). 2013;21(1):193‐202. 23505186 10.1002/oby.20243 PMC3666094

[78] Landgraf K, Klöting N, Gericke M, et al The obesity-susceptibility gene TMEM18 promotes adipogenesis through activation of PPARG. Cell Rep. 2020;33(3):108295. 33086065 10.1016/j.celrep.2020.108295

[79] Li J, Zhou L, Ouyang X, He P. Transcription factor-7-like-2 (TCF7L2) in Atherosclerosis: a potential biomarker and therapeutic target. Front Cardiovasc Med. 2021;8:701279. 34568447 10.3389/fcvm.2021.701279 PMC8459927

[80] Yang Y, Cao J, Shi Y. Identification and characterization of a gene encoding human LPGAT1, an endoplasmic reticulum-associated lysophosphatidylglycerol acyltransferase. J Biol Chem. 2004;279(53):55866‐55874. 15485873 10.1074/jbc.M406710200

[81] Little B . Human Biology: An introduction to Human Evolution, Variation, Growth, and Adaptability, by GA Harrison, JM Tanner, DR Pilbeam, and PT Baker. xv+ 568 pp. 3rd ed. Oxford University Press; 1988, $35.00 (paper).

[82] Hanson RL, Safabakhsh S, Curtis JM, et al Association of CREBRF variants with obesity and diabetes in Pacific Islanders from Guam and Saipan. Diabetologia. 2019;62(9):1647‐1652. 31280340 10.1007/s00125-019-4932-z PMC6721609

[83] Deka R, Xu L, Pal P, et al A tagging SNP in INSIG2 is associated with obesity-related phenotypes among Samoans. BMC Med Genet. 2009;10:143. 20028541 10.1186/1471-2350-10-143 PMC2804583

[84] Wu Y, Li C, Khan AA, et al Insulin-induced gene 2 protects against hepatic ischemia-reperfusion injury via metabolic remodeling. J Transl Med. 2023;21(1):739. 37858181 10.1186/s12967-023-04564-y PMC10585752

[85] Zhang K, Kaufman RJ. From endoplasmic-reticulum stress to the inflammatory response. Nature. 2008;454(7203):455‐462. 18650916 10.1038/nature07203 PMC2727659

[86] Loos RJ, Yeo GS. The bigger picture of FTO: the first GWAS-identified obesity gene. Nat Rev Endocrinol. 2014;10(1):51‐61. 24247219 10.1038/nrendo.2013.227 PMC4188449

[87] Loos RJ, Lindgren CM, Li S, et al Common variants near MC4R are associated with fat mass, weight and risk of obesity. Nat Genet. 2008;40(6):768‐775. 18454148 10.1038/ng.140 PMC2669167

[88] Gong J, Schumacher F, Lim U, et al Fine mapping and identification of BMI Loci in African Americans. Am J Hum Genet. 2013;93(4):661‐671. 24094743 10.1016/j.ajhg.2013.08.012 PMC3791273

[89] Olza J, Ruperez AI, Gil-Campos M, et al Influence of FTO variants on obesity, inflammation and cardiovascular disease risk biomarkers in Spanish children: a case-control multicentre study. BMC Med Genet. 2013;14:123. 24289790 10.1186/1471-2350-14-123 PMC3866940

[90] McFadden MJ, Sacco MT, Murphy KA, et al FTO suppresses STAT3 activation and modulates proinflammatory interferon-stimulated gene expression. J Mol Biol. 2022;434(6):167247. 34537236 10.1016/j.jmb.2021.167247 PMC8924017

[91] Xu ZY, Jing X, Xiong XD. Emerging role and mechanism of the *FTO* gene in cardiovascular diseases. Biomolecules. 2023;13(5):850. 37238719 10.3390/biom13050850 PMC10216201

[92] Gan X, Dai Z, Ge C, et al FTO promotes liver inflammation by suppressing m6A mRNA methylation of IL-17RA. Front Oncol. 2022;12:989353. 36172147 10.3389/fonc.2022.989353 PMC9511030

[93] Luo J, Wang F, Sun F, et al Targeted inhibition of FTO demethylase protects mice against LPS-induced septic shock by suppressing NLRP3 inflammasome. Front Immunol. 2021;12:663295. 34017338 10.3389/fimmu.2021.663295 PMC8128997

[94] Du J, Liao W, Liu W, et al N^6^-Adenosine methylation of Socs1 mRNA is required to sustain the negative feedback control of macrophage activation. Dev Cell. 2020;55(6):737‐753.e7. 33220174 10.1016/j.devcel.2020.10.023 PMC7755741

[95] Wu J, Wang X, Li X. N6-methyladenosine methylation regulator FTO promotes oxidative stress and induces cell apoptosis in ovarian cancer. Epigenomics. 2022;14(23):1509‐1522. 36815224 10.2217/epi-2022-0403

[96] Hu F, Tong J, Deng B, Zheng J, Lu C. MiR-495 regulates macrophage M1/M2 polarization and insulin resistance in high-fat diet-fed mice via targeting FTO. Pflugers Arch. 2019;471(11-12):1529‐1537. 31709454 10.1007/s00424-019-02316-w

[97] Xu M, Zhuo R, Tao S, et al M^6^a RNA methylation mediates NOD1/NF-kB signaling activation in the liver of piglets challenged with lipopolysaccharide. Antioxidants (Basel). 2022;11(10):1954. 36290677 10.3390/antiox11101954 PMC9598714

[98] Alipour M, Rostami H, Parastouei K. Association between inflammatory obesity phenotypes, FTO-rs9939609, and cardiovascular risk factors in patients with type 2 diabetes. J Res Med Sci. 2020;25:46. 32765616 10.4103/jrms.JRMS_429_19 PMC7377118

[99] Kamermans A, Verhoeven T, van Het Hof B, et al Setmelanotide, a novel, selective melanocortin receptor-4 agonist exerts anti-inflammatory actions in astrocytes and promotes an anti-inflammatory macrophage phenotype. Front Immunol. 2019;10:2312. 31636637 10.3389/fimmu.2019.02312 PMC6788433

[100] Konuma K, Itoh M, Suganami T, et al Eicosapentaenoic acid ameliorates non-alcoholic steatohepatitis in a novel mouse model using melanocortin 4 receptor-deficient mice. PLoS One. 2015;10(3):e0121528. 25816330 10.1371/journal.pone.0121528 PMC4376873

[101] Trevaskis JL, Gawronska-Kozak B, Sutton GM, et al Role of adiponectin and inflammation in insulin resistance of Mc3r and Mc4r knockout mice. Obesity (Silver Spring). 2007;15(11):2664‐2672. 18070757 10.1038/oby.2007.318 PMC2753182

[102] Malik IA, Triebel J, Posselt J, et al Melanocortin receptors in rat liver cells: change of gene expression and intracellular localization during acute-phase response. Histochem Cell Biol. 2012;137(3):279‐291. 22183812 10.1007/s00418-011-0899-7 PMC3312751

[103] Locke AE, Kahali B, Berndt SI, et al Genetic studies of body mass index yield new insights for obesity biology. Nature. 2015;518(7538):197‐206. 25673413 10.1038/nature14177 PMC4382211

[104] Sona C, Yeh YT, Patsalos A, et al Evidence of islet CADM1-mediated immune cell interactions during human type 1 diabetes. JCI Insight. 2022;7(6):e153136. 35133983 10.1172/jci.insight.153136 PMC8986082

[105] Baek YS, Haas S, Hackstein H, et al Identification of novel transcriptional regulators involved in macrophage differentiation and activation in U937 cells. BMC Immunol. 2009;10:18. 19341462 10.1186/1471-2172-10-18 PMC2674038

[106] Chen S, Xing Z, Geng M, et al Macrophage fusion event as one prerequisite for inorganic nanoparticle-induced antitumor response. Sci Adv. 2023;9(29):eadd9871. 37467339 10.1126/sciadv.add9871 PMC10355827

[107] Luo G, Zhou Z, Cao Z, et al M2 macrophage-derived exosomes induce angiogenesis and increase skin flap survival through HIF1AN/HIF-1α/VEGFA control. Arch Biochem Biophys. 2024;751:109822. 38030054 10.1016/j.abb.2023.109822

[108] Schumacher MA, Dennis IC, Liu CY, et al NRG4-ErbB4 signaling represses proinflammatory macrophage activity. Am J Physiol Gastrointest Liver Physiol. 2021;320(6):G990‐G1001. 33826403 10.1152/ajpgi.00296.2020 PMC8285586

[109] Zhao P, Hou N, Lu Y. Fhit protein is preferentially expressed in the nucleus of monocyte-derived cells and its possible biological significance. Histol Histopathol. 2006;21(9):915‐923. 16763940 10.14670/HH-21.915

[110] Black JA, Waxman SG. Noncanonical roles of voltage-gated sodium channels. Neuron. 2013;80(2):280‐291. 24139034 10.1016/j.neuron.2013.09.012

[111] Schönfelder J, Seibold T, Morawe M, et al Endothelial protein kinase D1 is a major regulator of post-traumatic hyperinflammation. Front Immunol. 2023;14:1093022. 36936923 10.3389/fimmu.2023.1093022 PMC10017463

[112] Ahmad S, Ahmed MM, Hasan PMZ, et al Identification and validation of potential miRNAs, as biomarkers for sepsis and associated lung injury: a network-based approach. Genes (Basel). 2020;11(11):1327. 33182754 10.3390/genes11111327 PMC7696689

[113] Xu J, Gao C, He Y, et al NLRC3 expression in macrophage impairs glycolysis and host immune defense by modulating the NF-κB-NFAT5 complex during septic immunosuppression. Mol Ther. 2023;31(1):154‐173. 36068919 10.1016/j.ymthe.2022.08.023 PMC9840117

[114] Poltorak A, He X, Smirnova I, et al Defective LPS signaling in C3H/HeJ and C57BL/10ScCr mice: mutations in Tlr4 gene. Science. 1998;282(5396):2085‐2088. 9851930 10.1126/science.282.5396.2085

[115] Huai W, Liu X, Wang C, et al KAT8 selectively inhibits antiviral immunity by acetylating IRF3. J Exp Med. 2019;216(4):772‐785. 30842237 10.1084/jem.20181773 PMC6446880

[116] Ma Y, Wang C, Xu G, et al Transcriptional changes in orthotopic liver transplantation and ischemia/reperfusion injury. Transpl Immunol. 2022;74:101638. 35667543 10.1016/j.trim.2022.101638

[117] Wei J, Dong S, Bowser RK, et al Regulation of the ubiquitylation and deubiquitylation of CREB-binding protein modulates histone acetylation and lung inflammation. Sci Signal. 2017;10(483):eaak9660. 28611184 10.1126/scisignal.aak9660 PMC5863726

[118] Antigny F, Hautefort A, Meloche J, et al Potassium channel subfamily K member 3 (KCNK3) contributes to the development of pulmonary arterial hypertension. Circulation. 2016;133(14):1371‐1385. 26912814 10.1161/CIRCULATIONAHA.115.020951

[119] Liang T, Zhu L, Yang J, et al Identification of key genes mediated by N6-methyladenosine methyltransferase METTL3 in ischemic stroke via bioinformatics analysis and experiments. Mol Biotechnol. 2023. Published online December 22, 2023. Doi:10.1007/s12033-023-00991-w 38135832

[120] Bissonnette S, Lamantia V, Ouimet B, et al Native low-density lipoproteins are priming signals of the NLRP3 inflammasome/interleukin-1β pathway in human adipose tissue and macrophages. Sci Rep. 2023;13(1):18848. 37914804 10.1038/s41598-023-45870-1 PMC10620147

[121] Ovsyannikova IG, Dhiman N, Haralambieva IH, et al Rubella vaccine-induced cellular immunity: evidence of associations with polymorphisms in the toll-like, vitamin A and D receptors, and innate immune response genes. Hum Genet. 2010;127(2):207‐221. 19902255 10.1007/s00439-009-0763-1 PMC2809817

[122] Chopra R, Kalaiarasan P, Ali S, et al PARK2 and proinflammatory/anti-inflammatory cytokine gene interactions contribute to the susceptibility to leprosy: a case-control study of north Indian population. BMJ Open. 2014;4(2):e004239. 10.1136/bmjopen-2013-004239 PMC3939656 24578538

[123] Zou J . The transcriptional profiling identifies hub genes in immune subsets of patients with Behçet's syndrome. Clin Exp Rheumatol. 2023;41(10):1955‐1963. 36226612 10.55563/clinexprheumatol/z4z0uj

[124] Baranova IN, Souza AC, Bocharov AV, et al Human SR-BI and SR-BII potentiate lipopolysaccharide-induced inflammation and acute liver and kidney injury in mice. J Immunol. 2016;196(7):3135‐3147. 26936883 10.4049/jimmunol.1501709 PMC4856165

[125] Zhang W, Wang YD, Xing YJ, Liu PJ, Yang JH. Silencing of circ-NT5C2 retards the progression of IL-1β-induced osteoarthritis in an in vitro cell model by targeting the miR-142-5p/NAMPT axis. Microbiol Immunol. 2023;67(3):129‐141. 36540014 10.1111/1348-0421.13046

[126] Arora H, Wilcox SM, Johnson LA, et al The ATP-binding cassette gene ABCF1 functions as an E2 ubiquitin-conjugating enzyme controlling macrophage polarization to dampen lethal septic shock. Immunity. 2019;50(2):418‐431.e6. 30770245 10.1016/j.immuni.2019.01.014

[127] Novikova G, Kapoor M, Tcw J, et al Integration of Alzheimer's disease genetics and myeloid genomics identifies disease risk regulatory elements and genes. Nat Commun. 2021;12(1):1610. 33712570 10.1038/s41467-021-21823-y PMC7955030

[128] Li J, Zhou H, Wei B, et al The rs8506 TT genotype in *lincRNA-NR_024015* contributes to the risk of sepsis in a southern Chinese child population. Front Public Health. 2022;10:927527. 35910890 10.3389/fpubh.2022.927527 PMC9326103

[129] Dorrity TJ, Shin H, Wiegand KA, et al Long 3'UTRs predispose neurons to inflammation by promoting immunostimulatory double-stranded RNA formation. Sci Immunol. 2023;8(88):eadg2979. 37862432 10.1126/sciimmunol.adg2979 PMC11056275

[130] Zhang S, Li Z, Weinman S. Foxo3 might be involved in the inflammatory response of human monocytes to lipopolysaccharide through regulating expression of toll like receptor 4. Mol Biol Rep. 2022;49(8):7611‐7621. 35618937 10.1007/s11033-022-07576-x PMC10829848

[131] Mu Y, Wang L, Fu L, Li Q. Knockdown of LMX1B suppressed cell apoptosis and inflammatory response in IL-1*β*-induced human osteoarthritis chondrocytes through NF-*κ*B and NLRP3 signal pathway. Mediators Inflamm. 2022;2022:1870579. 36133743 10.1155/2022/1870579 PMC9484960

[132] Jakka P, Bhargavi B, Namani S, Murugan S, Splitter G, Radhakrishnan G. Cytoplasmic linker protein CLIP170 negatively regulates TLR4 signaling by targeting the TLR adaptor protein TIRAP. J Immunol. 2018;200(2):704‐714. 29222167 10.4049/jimmunol.1601559 PMC5760445

[133] Yun JH, Lee C, Liu T, et al Hedgehog interacting protein-expressing lung fibroblasts suppress lymphocytic inflammation in mice. JCI Insight. 2021;6(17):e144575. 34375314 10.1172/jci.insight.144575 PMC8492352

[134] Heikelä H, Ruohonen ST, Adam M, et al Hydroxysteroid (17β) dehydrogenase 12 is essential for metabolic homeostasis in adult mice. Am J Physiol Endocrinol Metab. 2020;319(3):E494‐E508. 32691632 10.1152/ajpendo.00042.2020

[135] Li H, Tang D, Chen J, Hu Y, Cai X, Zhang P. The clinical value of GDF15 and its prospective mechanism in sepsis. Front Immunol. 2021;12:710977. 34566964 10.3389/fimmu.2021.710977 PMC8456026

[136] Viegas CSB, Costa RM, Santos L, et al Gla-rich protein function as an anti-inflammatory agent in monocytes/macrophages: implications for calcification-related chronic inflammatory diseases. PLoS One. 2017;12(5):e0177829. 28542410 10.1371/journal.pone.0177829 PMC5436823

[137] Liang Z, Rehati A, Husaiyin E, Chen D, Jiyuan Z, Abuduaini B. RALY regulate the proliferation and expression of immune/inflammatory response genes via alternative splicing of FOS. Genes Immun. 2022;23(8):246‐254. 35941292 10.1038/s41435-022-00178-4 PMC9758052

[138] Duncan BB, Schmidt MI, Chambless LE, Folsom AR, Carpenter M, Heiss G. Fibrinogen, other putative markers of inflammation, and weight gain in middle-aged adults–the ARIC study. Atherosclerosis risk in communities. Obes Res. 2000;8:279‐286. 10933303 10.1038/oby.2000.33

[139] Holz T, Thorand B, Döring A, Schneider A, Meisinger C, Koenig W. Markers of inflammation and weight change in middle-aged adults: results from the prospective MONICA/KORA S3/F3 study. Obesity (Silver Spring). 2010;18(12):2347‐2353. 20360759 10.1038/oby.2010.73

[140] Mori MA, Liu M, Bezy O, et al A systems biology approach identifies inflammatory abnormalities between mouse strains prior to development of metabolic disease. Diabetes. 2010;59(11):2960‐2971. 20713682 10.2337/db10-0367 PMC2963557

[141] Prada PO, Zecchin HG, Gasparetti AL, et al Western diet modulates insulin signaling, c-Jun N-terminal kinase activity, and insulin receptor substrate-1ser307 phosphorylation in a tissue-specific fashion. Endocrinology. 2005;146(3):1576‐1587. 15591151 10.1210/en.2004-0767

[142] Abboud KY, Reis SK, Martelli ME, et al Oral glutamine supplementation reduces obesity, pro-inflammatory markers, and improves insulin sensitivity in DIO wistar rats and reduces waist circumference in overweight and obese humans. Nutrients. 2019;11(3):536. 30832230 10.3390/nu11030536 PMC6471297

[143] Saad MJ, Araki E, Miralpeix M, Rothenberg PL, White MF, Kahn CR. Regulation of insulin receptor substrate-1 in liver and muscle of animal models of insulin resistance. J Clin Invest. 1992;90(5):1839‐1849. 1331176 10.1172/JCI116060 PMC443244

[144] Zanotto TM, Quaresma PGF, Guadagnini D, et al Blocking iNOS and endoplasmic reticulum stress synergistically improves insulin resistance in mice. Mol Metab. 2017;6(2):206‐218. 28180062 10.1016/j.molmet.2016.12.005 PMC5279911

[145] Zou W, Rohatgi N, Brestoff JR, et al Myeloid-specific Asxl2 deletion limits diet-induced obesity by regulating energy expenditure. J Clin Invest. 2020;130(5):2644‐2656. 32310225 10.1172/JCI128687 PMC7190927

[146] Rajasekaran M, Sul OJ, Choi EK, Kim JE, Suh JH, Choi HS. MCP-1 deficiency enhances browning of adipose tissue via increased M2 polarization. J Endocrinol. 2019;242(2):91‐101. 31137011 10.1530/JOE-19-0190

[147] Ferreira SRG, Macotela Y, Velloso LA, Mori MA. Determinants of obesity in Latin America. Nat Metab. 2024;6(3):409‐432. 38438626 10.1038/s42255-024-00977-1

[148] Levitsky DA, Pacanowski CR. Free will and the obesity epidemic. Public Health Nutr. 2012;15(1):126‐141. 21923977 10.1017/S1368980011002187

[149] Monteiro CA, Conde WL, Popkin BM. Independent effects of income and education on the risk of obesity in the Brazilian adult population. J Nutr. 2001;131(3):881S‐886S. 11238779 10.1093/jn/131.3.881S

[150] Bell ML, Davis DL, Gouveia N, Borja-Aburto VH, Cifuentes LA. The avoidable health effects of air pollution in three Latin American cities: Santiago, São Paulo, and Mexico city. Environ Res. 2006;100(3):431‐440. 16181621 10.1016/j.envres.2005.08.002

[151] Souza MCO, Rocha BA, Adeyemi JA, et al Legacy and emerging pollutants in Latin America: a critical review of occurrence and levels in environmental and food samples. Sci Total Environ. 2022;848:157774. 35932867 10.1016/j.scitotenv.2022.157774

[152] Myers S, Fanzo J, Wiebe K, Huybers P, Smith M. Current guidance underestimates risk of global environmental change to food security. BMJ. 2022;378:e071533. 36175018 10.1136/bmj-2022-071533 PMC9517947

[153] Yang W, Kelly T, He J. Genetic epidemiology of obesity. Epidemiol Rev. 2007;29:49‐61. 17566051 10.1093/epirev/mxm004

[154] Wells JC, Marphatia AA, Cole TJ, McCoy D. Associations of economic and gender inequality with global obesity prevalence: understanding the female excess. Soc Sci Med. 2012;75(3):482‐490. 22580078 10.1016/j.socscimed.2012.03.029

[155] Alberdi A, Aizpurua O, Bohmann K, Zepeda-Mendoza ML, Gilbert MTP. Do vertebrate gut metagenomes confer rapid ecological adaptation? Trends Ecol Evol. 2016;31(9):689‐699. 27453351 10.1016/j.tree.2016.06.008

[156] Shapira M . Gut microbiotas and host evolution: scaling up symbiosis. Trends Ecol Evol. 2016;31(7):539‐549. 27039196 10.1016/j.tree.2016.03.006

[157] Suzuki TA, Ley RE. The role of the microbiota in human genetic adaptation. Science. 2020;370(6521):eaaz6827. 33273073 10.1126/science.aaz6827

[158] Quan Y, Zhang KX, Zhang HY. The gut microbiota links disease to human genome evolution. Trends Genet. 2023;39(6):451‐461. 36872184 10.1016/j.tig.2023.02.006

[159] Rook G, Bäckhed F, Levin BR, McFall-Ngai MJ, McLean AR. Evolution, human-microbe interactions, and life history plasticity. Lancet. 2017;390(10093):521‐530. 28792414 10.1016/S0140-6736(17)30566-4

[160] Bäckhed F, Ley RE, Sonnenburg JL, Peterson DA, Gordon JI. Host-bacterial mutualism in the human intestine. Science. 2005;307(5717):1915‐1920. 15790844 10.1126/science.1104816

[161] Garrett WS, Gordon JI, Glimcher LH. Homeostasis and inflammation in the intestine. Cell. 2010;140(6):859‐870. 20303876 10.1016/j.cell.2010.01.023 PMC2845719

[162] Tremaroli V, Bäckhed F. Functional interactions between the gut microbiota and host metabolism. Nature. 2012;489(7415):242‐249. 22972297 10.1038/nature11552

[163] Wu H, Tremaroli V, Bäckhed F. Linking microbiota to human diseases: a systems biology perspective. Trends Endocrinol Metab. 2015;26(12):758‐770. 26555600 10.1016/j.tem.2015.09.011

[164] Lozupone CA, Stombaugh JI, Gordon JI, Jansson JK, Knight R. Diversity, stability and resilience of the human gut microbiota. Nature. 2012;489(7415):220‐230. 22972295 10.1038/nature11550 PMC3577372

[165] Greiner T, Bäckhed F. Effects of the gut microbiota on obesity and glucose homeostasis. Trends Endocrinol Metab. 2011;22(4):117‐123. 21353592 10.1016/j.tem.2011.01.002

[166] Sommer F, Bäckhed F. Know your neighbor: microbiota and host epithelial cells interact locally to control intestinal function and physiology. Bioessays. 2016;38(5):455‐464. 26990415 10.1002/bies.201500151

[167] Sonnenburg JL, Bäckhed F. Diet-microbiota interactions as moderators of human metabolism. Nature. 2016;535(7610):56‐64. 27383980 10.1038/nature18846 PMC5991619

[168] Mei Z, Wang F, Bhosle A, et al Strain-specific gut microbial signatures in type 2 diabetes identified in a cross-cohort analysis of 8,117 metagenomes. Nat Med. 2024;30(8):2265‐2276. 38918632 10.1038/s41591-024-03067-7 PMC11620793

[169] Chen S, Tu M, Shi J, Hu X. Changes of intestinal flora in patients with atrial fibrillation and its correlation with cardiovascular risk factors. Rev Cardiovasc Med. 2023;24(4):110. 39076278 10.31083/j.rcm2404110 PMC11273065

[170] Schroeder BO, Bäckhed F. Signals from the gut microbiota to distant organs in physiology and disease. Nat Med. 2016;22(10):1079‐1089. 27711063 10.1038/nm.4185

[171] Clemente JC, Ursell LK, Parfrey LW, Knight R. The impact of the gut microbiota on human health: an integrative view. Cell. 2012;148(6):1258‐1270. 22424233 10.1016/j.cell.2012.01.035 PMC5050011

[172] Gilbert JA, Blaser MJ, Caporaso JG, Jansson JK, Lynch SV, Knight R. Current understanding of the human microbiome. Nat Med. 2018;24(4):392‐400. 29634682 10.1038/nm.4517 PMC7043356

[173] Ferreiro A, Crook N, Gasparrini AJ, Dantas G. Multiscale evolutionary dynamics of host-associated microbiomes. Cell. 2018;172(6):1216‐1227. 29522743 10.1016/j.cell.2018.02.015 PMC5846202

[174] Lee YK, Mazmanian SK. Has the microbiota played a critical role in the evolution of the adaptive immune system? Science. 2010;330(6012):1768‐1773. 21205662 10.1126/science.1195568 PMC3159383

[175] David LA, Maurice CF, Carmody RN, et al Diet rapidly and reproducibly alters the human gut microbiome. Nature. 2014;505(7484):559‐563. 24336217 10.1038/nature12820 PMC3957428

[176] De Filippo C, Cavalieri D, Di Paola M, et al Impact of diet in shaping gut microbiota revealed by a comparative study in children from Europe and rural Africa. Proc Natl Acad Sci U S A. 2010;107(33):14691‐14696. 20679230 10.1073/pnas.1005963107 PMC2930426

[177] Wu GD, Chen J, Hoffmann C, et al Linking long-term dietary patterns with gut microbial enterotypes. Science. 2011;334(6052):105‐108. 21885731 10.1126/science.1208344 PMC3368382

[178] Cotillard A, Kennedy SP, Kong LC, et al Dietary intervention impact on gut microbial gene richness. Nature. 2013;500(7464):585‐588. 23985875 10.1038/nature12480

[179] Kovatcheva-Datchary P, Nilsson A, Akrami R, et al Dietary fiber-induced improvement in glucose metabolism is associated with increased abundance of Prevotella. Cell Metab. 2015;22(6):971‐982. 26552345 10.1016/j.cmet.2015.10.001

[180] Walker AW, Ince J, Duncan SH, et al Dominant and diet-responsive groups of bacteria within the human colonic microbiota. ISME J. 2011;5(2):220‐230. 20686513 10.1038/ismej.2010.118 PMC3105703

[181] Ley RE, Hamady M, Lozupone C, et al Evolution of mammals and their gut microbes. Science. 2008;320(5883):1647‐1651. 18497261 10.1126/science.1155725 PMC2649005

[182] Muegge BD, Kuczynski J, Knights D, et al Diet drives convergence in gut microbiome functions across mammalian phylogeny and within humans. Science. 2011;332(6032):970‐974. 21596990 10.1126/science.1198719 PMC3303602

[183] Magro DO, Rossoni C, Saad-Hossne R, Santos A. Interaction between food pyramid and gut microbiota. A new nutritional approach. Arq Gastroenterol. 2023;60(1):132‐136. 37194771 10.1590/S0004-2803.202301000-15

[184] Clayton JB, Gomez A, Amato K, et al The gut microbiome of nonhuman primates: lessons in ecology and evolution. Am J Primatol. 2018;80(6):e22867. 29862519 10.1002/ajp.22867

[185] Wastyk HC, Fragiadakis GK, Perelman D, et al Gut-microbiota-targeted diets modulate human immune status. Cell. 2021;184(16):4137‐4153.e14. 34256014 10.1016/j.cell.2021.06.019 PMC9020749

[186] Thaiss CA, Zeevi D, Levy M, et al Transkingdom control of microbiota diurnal oscillations promotes metabolic homeostasis. Cell. 2014;159(3):514‐529. 25417104 10.1016/j.cell.2014.09.048

[187] Tuganbaev T, Mor U, Bashiardes S, et al Diet diurnally regulates small intestinal microbiome-epithelial-immune homeostasis and enteritis. Cell. 2020;182(6):1441‐1459.e21. 32888430 10.1016/j.cell.2020.08.027

[188] Yatsunenko T, Rey FE, Manary MJ, et al Human gut microbiome viewed across age and geography. Nature. 2012;486(7402):222‐227. 22699611 10.1038/nature11053 PMC3376388

[189] Hehemann JH, Correc G, Barbeyron T, Helbert W, Czjzek M, Michel G. Transfer of carbohydrate-active enzymes from marine bacteria to Japanese gut microbiota. Nature. 2010;464(7290):908‐912. 20376150 10.1038/nature08937

[190] Groussin M, Poyet M, Sistiaga A, et al Elevated rates of horizontal gene transfer in the industrialized human microbiome. Cell. 2021;184(8):2053‐2067.e18. 33794144 10.1016/j.cell.2021.02.052

[191] Gacesa R, Kurilshikov A, Vich Vila A, et al Environmental factors shaping the gut microbiome in a Dutch population. Nature. 2022;604(7907):732‐739. 35418674 10.1038/s41586-022-04567-7

[192] Cox LM, Yamanishi S, Sohn J, et al Altering the intestinal microbiota during a critical developmental window has lasting metabolic consequences. Cell. 2014;158(4):705‐721. 25126780 10.1016/j.cell.2014.05.052 PMC4134513

[193] Seeman MV . The gut microbiome and antipsychotic treatment response. Behav Brain Res. 2021;396:112886. 32890599 10.1016/j.bbr.2020.112886

[194] Maier L, Pruteanu M, Kuhn M, et al Extensive impact of non-antibiotic drugs on human gut bacteria. Nature. 2018;555(7698):623‐628. 29555994 10.1038/nature25979 PMC6108420

[195] Fan Y, Pedersen O. Gut microbiota in human metabolic health and disease. Nat Rev Microbiol. 2021;19(1):55‐71. 32887946 10.1038/s41579-020-0433-9

[196] Zhao L . The gut microbiota and obesity: from correlation to causality. Nat Rev Microbiol. 2013;11(9):639‐647. 23912213 10.1038/nrmicro3089

[197] Wang J, Jia H. Metagenome-wide association studies: fine-mining the microbiome. Nat Rev Microbiol. 2016;14(8):508‐522. 27396567 10.1038/nrmicro.2016.83

[198] Van Hul M, Cani PD. The gut microbiota in obesity and weight management: microbes as friends or foe? Nat Rev Endocrinol. 2023;19(5):258‐271. 36650295 10.1038/s41574-022-00794-0

[199] Mayneris-Perxachs J, Moreno-Navarrete JM, Fernández-Real JM. The role of iron in host-microbiota crosstalk and its effects on systemic glucose metabolism. Nat Rev Endocrinol. 2022;18(11):683‐698. 35986176 10.1038/s41574-022-00721-3

[200] Bishehsari F, Voigt RM, Keshavarzian A. Circadian rhythms and the gut microbiota: from the metabolic syndrome to cancer. Nat Rev Endocrinol. 2020;16(12):731‐739. 33106657 10.1038/s41574-020-00427-4 PMC8085809

[201] Canfora EE, Meex RCR, Venema K, Blaak EE. Gut microbial metabolites in obesity, NAFLD and T2DM. Nat Rev Endocrinol. 2019;15(5):261‐273. 30670819 10.1038/s41574-019-0156-z

[202] Cani PD . Microbiota and metabolites in metabolic diseases. Nat Rev Endocrinol. 2019;15(2):69‐70. 30602737 10.1038/s41574-018-0143-9

[203] Greenhill C . Obesity: gut microbiota, host genetics and diet interact to affect the risk of developing obesity and the metabolic syndrome. Nat Rev Endocrinol. 2015;11(11):630. 10.1038/nrendo.2015.152 26346953

[204] Ray K . Gut microbiota: adding weight to the microbiota's role in obesity–exposure to antibiotics early in life can lead to increased adiposity. Nat Rev Endocrinol. 2012;8(11):623. 22965166 10.1038/nrendo.2012.173

[205] Cani PD, Van Hul M. Gut microbiota in overweight and obesity: crosstalk with adipose tissue. Nat Rev Gastroenterol Hepatol. 2024;21(3):164‐183. 38066102 10.1038/s41575-023-00867-z

[206] Zhou Z, Sun B, Yu D, Zhu C. Gut microbiota: an important player in type 2 diabetes mellitus. Front Cell Infect Microbiol. 2022;12:834485. 35242721 10.3389/fcimb.2022.834485 PMC8886906

[207] Withrow D, Bowers SJ, Depner CM, González A, Reynolds AC, Wright KP. Sleep and circadian disruption and the gut microbiome-possible links to dysregulated metabolism. Curr Opin Endocr Metab Res. 2021;17:26‐37. 34805616 10.1016/j.coemr.2020.11.009 PMC8597978

[208] Goodrich JK, Waters JL, Poole AC, et al Human genetics shape the gut microbiome. Cell. 2014;159(4):789‐799. 25417156 10.1016/j.cell.2014.09.053 PMC4255478

[209] Goodrich JK, Davenport ER, Beaumont M, et al Genetic determinants of the gut microbiome in UK twins. Cell Host Microbe. 2016;19(5):731‐743. 27173935 10.1016/j.chom.2016.04.017 PMC4915943

[210] Zhernakova DV, Wang D, Liu L, et al Host genetic regulation of human gut microbial structural variation. Nature. 2024;625(7996):813‐821. 38172637 10.1038/s41586-023-06893-w PMC10808065

[211] Zhang CY, Jiang SJ, Cao JJ, et al Investigating the causal relationship between gut microbiota and gastroenteropancreatic neuroendocrine neoplasms: a bidirectional Mendelian randomization study. Front Microbiol. 2024;15:1420167. 39193433 10.3389/fmicb.2024.1420167 PMC11347282

[212] Zampieri G, Cabrol L, Urra C, et al Microbiome alterations are associated with apolipoprotein E mutation in *Octodon degus* and humans with Alzheimer's disease. iScience. 2024;27(8):110348. 39148714 10.1016/j.isci.2024.110348 PMC11324989

[213] Jiang P, Li C, Su Z, et al Mendelian randomization study reveals causal effects of specific gut microbiota on the risk of interstitial cystitis/bladder pain syndrome (IC/BPS). Sci Rep. 2024;14(1):18405. 39117770 10.1038/s41598-024-69543-9 PMC11310512

[214] Wekema L, Schoenmakers S, Schenkelaars N, et al Obesity and diet independently affect maternal immunity, maternal gut microbiota and pregnancy outcome in mice. Front Immunol. 2024;15:1376583. 39072322 10.3389/fimmu.2024.1376583 PMC11272480

[215] Ley RE, Bäckhed F, Turnbaugh P, Lozupone CA, Knight RD, Gordon JI. Obesity alters gut microbial ecology. Proc Natl Acad Sci U S A. 2005;102(31):11070‐11075. 16033867 10.1073/pnas.0504978102 PMC1176910

[216] Ley RE, Turnbaugh PJ, Klein S, Gordon JI. Microbial ecology: human gut microbes associated with obesity. Nature. 2006;444(7122):1022‐1023. 17183309 10.1038/4441022a

[217] Backhed F, Manchester JK, Semenkovich CF, Gordon JI. Mechanisms underlying the resistance to diet-induced obesity in germ-free mice. Proc Natl Acad Sci U S A. 2007;104(3):979‐984. 17210919 10.1073/pnas.0605374104 PMC1764762

[218] Bäckhed F, Ding H, Wang T, et al The gut microbiota as an environmental factor that regulates fat storage. Proc Natl Acad Sci U S A. 2004;101(44):15718‐15723. 15505215 10.1073/pnas.0407076101 PMC524219

[219] Turnbaugh PJ, Ley RE, Mahowald MA, Magrini V, Mardis ER, Gordon JI. An obesity-associated gut microbiome with increased capacity for energy harvest. Nature. 2006;444(7122):1027‐1031. 17183312 10.1038/nature05414

[220] Vijay-Kumar M, Aitken JD, Carvalho FA, et al Metabolic syndrome and altered gut microbiota in mice lacking toll-like receptor 5. Science. 2010;328(5975):228‐231. 20203013 10.1126/science.1179721 PMC4714868

[221] Ussar S, Griffin NW, Bezy O, et al Interactions between gut microbiota, host genetics and diet modulate the predisposition to obesity and metabolic syndrome. Cell Metab. 2015;22(3):516‐530. 26299453 10.1016/j.cmet.2015.07.007 PMC4570502

[222] Guadagnini D, Rocha GZ, Santos A, et al Microbiota determines insulin sensitivity in TLR2-KO mice. Life Sci. 2019;234:116793. 31465735 10.1016/j.lfs.2019.116793

[223] Peters BA, Shapiro JA, Church TR, et al A taxonomic signature of obesity in a large study of American adults. Sci Rep. 2018;8(1):9749. 29950689 10.1038/s41598-018-28126-1 PMC6021409

[224] Palmas V, Pisanu S, Madau V, et al Gut microbiota markers associated with obesity and overweight in Italian adults. Sci Rep. 2021;11(1):5532. 33750881 10.1038/s41598-021-84928-w PMC7943584

[225] Gao X, Jia R, Xie L, Kuang L, Feng L, Wan C. A study of the correlation between obesity and intestinal flora in school-age children. Sci Rep. 2018;8(1):14511. 30267022 10.1038/s41598-018-32730-6 PMC6162261

[226] Romanini E, Padua R, Tucci G, Zanoli G; Gruppo di Lavoro Ortopedia Basata su prove di Efficacia. Ortopedia tra ragione e passione. Linee guida e linee d’ombra [orthopedics between reason and passion. Guidelines and shadow lines.]. Recenti Prog Med. 2020;111(6):354‐356. 32573550 10.1701/3394.33756

[227] Finucane MM, Sharpton TJ, Laurent TJ, Pollard KS. A taxonomic signature of obesity in the microbiome? Getting to the guts of the matter. PLoS One. 2014;9(1):e84689. 24416266 10.1371/journal.pone.0084689 PMC3885756

[228] Armour CR, Nayfach S, Pollard KS, Sharpton TJ. A metagenomic meta-analysis reveals functional signatures of health and disease in the human gut microbiome. mSystems. 2019;4(4):e00332-18. 31098399 10.1128/mSystems.00332-18 PMC6517693

[229] Del Chierico F, Abbatini F, Russo A, et al Gut microbiota markers in obese adolescent and adult patients: age-dependent differential patterns. Front Microbiol. 2018;9:1210. 29922272 10.3389/fmicb.2018.01210 PMC5996250

[230] Stanislawski MA, Dabelea D, Lange LA, Wagner BD, Lozupone CA. Gut microbiota phenotypes of obesity. NPJ Biofilms Microbiomes. 2019;5(1):18. 31285833 10.1038/s41522-019-0091-8 PMC6603011

[231] Turnbaugh PJ, Hamady M, Yatsunenko T, et al A core gut microbiome in obese and lean twins. Nature. 2009;457(7228):480‐484. 19043404 10.1038/nature07540 PMC2677729

[232] Armougom F, Henry M, Vialettes B, Raccah D, Raoult D. Monitoring bacterial community of human gut microbiota reveals an increase in Lactobacillus in obese patients and methanogens in anorexic patients. PLoS One. 2009;4(9):e7125. 19774074 10.1371/journal.pone.0007125 PMC2742902

[233] Schwiertz A, Taras D, Schäfer K, et al Microbiota and SCFA in lean and overweight healthy subjects. Obesity (Silver Spring). 2010;18(1):190‐195. 19498350 10.1038/oby.2009.167

[234] Mai V, McCrary QM, Sinha R, Glei M. Associations between dietary habits and body mass index with gut microbiota composition and fecal water genotoxicity: an observational study in African American and Caucasian American volunteers. Nutr J. 2009;8:49. 19845958 10.1186/1475-2891-8-49 PMC2773807

[235] Duncan SH, Lobley GE, Holtrop G, et al Human colonic microbiota associated with diet, obesity and weight loss. Int J Obes (Lond). 2008;32(11):1720‐1724. 18779823 10.1038/ijo.2008.155

[236] Tims S, Derom C, Jonkers DM, et al Microbiota conservation and BMI signatures in adult monozygotic twins. ISME J. 2013;7(4):707‐717. 23190729 10.1038/ismej.2012.146 PMC3603393

[237] Yun Y, Kim HN, Kim SE, et al Comparative analysis of gut microbiota associated with body mass index in a large Korean cohort. BMC Microbiol. 2017;17(1):151. 28676106 10.1186/s12866-017-1052-0 PMC5497371

[238] Walters WA, Xu Z, Knight R. Meta-analyses of human gut microbes associated with obesity and IBD. FEBS Lett. 2014;588(22):4223‐4233. 25307765 10.1016/j.febslet.2014.09.039 PMC5050012

[239] Sze MA, Schloss PD. Looking for a signal in the noise: revisiting obesity and the microbiome. mBio. 2016;7(4):e01018-16. 27555308 10.1128/mBio.01018-16 PMC4999546

[240] Pinart M, Dötsch A, Schlicht K, et al Gut microbiome composition in obese and non-obese persons: a systematic review and meta-analysis. Nutrients. 2021;14(1):12. 35010887 10.3390/nu14010012 PMC8746372

[241] Xu Z, Jiang W, Huang W, Lin Y, Chan FKL, Ng SC. Gut microbiota in patients with obesity and metabolic disorders—a systematic review. Genes Nutr. 2022;17(1):2. 35093025 10.1186/s12263-021-00703-6 PMC8903526

[242] Crovesy L, Masterson D, Rosado EL. Profile of the gut microbiota of adults with obesity: a systematic review. Eur J Clin Nutr. 2020;74(9):1251‐1262. 32231226 10.1038/s41430-020-0607-6

[243] Thingholm LB, Rühlemann MC, Koch M, et al Obese individuals with and without type 2 diabetes show different gut microbial functional capacity and composition. Cell Host Microbe. 2019;26(2):252‐264.e10. 31399369 10.1016/j.chom.2019.07.004 PMC7720933

[244] Duarte SMB, Stefano JT, Miele L, et al Gut microbiome composition in lean patients with NASH is associated with liver damage independent of caloric intake: a prospective pilot study. Nutr Metab Cardiovasc Dis. 2018;28(4):369‐384. 29482963 10.1016/j.numecd.2017.10.014

[245] Zahavi L, Lavon A, Reicher L, et al Bacterial SNPs in the human gut microbiome associate with host BMI. Nat Med. 2023;29(11):2785‐2792. 37919437 10.1038/s41591-023-02599-8 PMC10999242

[246] Bordenstein SR, Theis KR. Host biology in light of the microbiome: ten principles of holobionts and hologenomes. PLoS Biol. 2015;13(8):e1002226. 26284777 10.1371/journal.pbio.1002226 PMC4540581

[247] Hurst GDD . Extended genomes: symbiosis and evolution. Interface Focus. 2017;7(5):20170001. 28839925 10.1098/rsfs.2017.0001 PMC5566813

[248] Rosenberg E, Zilber-Rosenberg I. The hologenome concept of evolution after 10 years. Microbiome. 2018;6(1):78. 29695294 10.1186/s40168-018-0457-9 PMC5922317

[249] Moeller AH, Suzuki TA, Phifer-Rixey M, Nachman MW. Transmission modes of the mammalian gut microbiota. Science. 2018;362(6413):453‐457. 30361372 10.1126/science.aat7164

[250] Walters WA, Jin Z, Youngblut N, et al Large-scale replicated field study of maize rhizosphere identifies heritable microbes. Proc Natl Acad Sci U S A. 2018;115(28):7368‐7373. 29941552 10.1073/pnas.1800918115 PMC6048482

[251] Camarinha-Silva A, Maushammer M, Wellmann R, Vital M, Preuss S, Bennewitz J. Host genome influence on gut microbial composition and microbial prediction of complex traits in pigs. Genetics. 2017;206(3):1637‐1644. 28468904 10.1534/genetics.117.200782 PMC5500156

[252] Elena SF, Lenski RE. Evolution experiments with microorganisms: the dynamics and genetic bases of adaptation. Nat Rev Genet. 2003;4(6):457‐469. 12776215 10.1038/nrg1088

[253] Henry LP, Bruijning M, Forsberg SKG, Ayroles JF. The microbiome extends host evolutionary potential. Nat Commun. 2021;12(1):5141. 34446709 10.1038/s41467-021-25315-x PMC8390463

[254] Sommer F, Ståhlman M, Ilkayeva O, et al The gut microbiota modulates energy metabolism in the hibernating brown bear Ursus arctos. Cell Rep. 2016;14(7):1655‐1661. 26854221 10.1016/j.celrep.2016.01.026

[255] McLaren MR, Callahan BJ. Pathogen resistance may be the principal evolutionary advantage provided by the microbiome. Philos Trans R Soc Lond B Biol Sci. 2020;375(1808):20190592. 32772671 10.1098/rstb.2019.0592 PMC7435163

[256] Bercik P, Denou E, Collins J, et al The intestinal microbiota affect central levels of brain-derived neurotropic factor and behavior in mice. Gastroenterology. 2011;141(2):e591‐609.e3. 10.1053/j.gastro.2011.04.052 21683077

[257] Bravo JA, Forsythe P, Chew MV, et al Ingestion of Lactobacillus strain regulates emotional behavior and central GABA receptor expression in a mouse via the vagus nerve. Proc Natl Acad Sci U S A. 2011;108(38):16050‐16055. 21876150 10.1073/pnas.1102999108 PMC3179073

[258] Cani PD, Amar J, Iglesias MA, et al Metabolic endotoxemia initiates obesity and insulin resistance. Diabetes. 2007;56(7):1761‐1772. 17456850 10.2337/db06-1491

[259] Cani PD, Bibiloni R, Knauf C, et al Changes in gut microbiota control metabolic endotoxemia-induced inflammation in high-fat diet-induced obesity and diabetes in mice. Diabetes. 2008;57(6):1470‐1481. 18305141 10.2337/db07-1403

[260] Carvalho BM, Guadagnini D, Tsukumo DML, et al Modulation of gut microbiota by antibiotics improves insulin signalling in high-fat fed mice. Diabetologia. 2012;55(10):2823‐2834. 22828956 10.1007/s00125-012-2648-4

[261] Carvalho-Filho MA, Carvalho BM, Oliveira AG, et al Double-stranded RNA-activated protein kinase is a key modulator of insulin sensitivity in physiological conditions and in obesity in mice. Endocrinology. 2012;153(11):5261‐5274. 22948222 10.1210/en.2012-1400

[262] Chevalier C, Stojanović O, Colin DJ, et al Gut microbiota orchestrates energy homeostasis during cold. Cell. 2015;163(6):1360‐1374. 26638070 10.1016/j.cell.2015.11.004

[263] Ziętak M, Kovatcheva-Datchary P, Markiewicz LH, Ståhlman M, Kozak LP, Bäckhed F. Altered microbiota contributes to reduced diet-induced obesity upon cold exposure. Cell Metab. 2016;23(6):1216‐1223. 27304513 10.1016/j.cmet.2016.05.001 PMC4911343

[264] Wang Z, Wu Y, Li X, Ji X, Liu W. The gut microbiota facilitate their host tolerance to extreme temperatures. BMC Microbiol. 2024;24(1):131. 38643098 10.1186/s12866-024-03277-6 PMC11031955

[265] Worthmann A, John C, Rühlemann MC, et al Cold-induced conversion of cholesterol to bile acids in mice shapes the gut microbiome and promotes adaptive thermogenesis. Nat Med. 2017;23(7):839‐849. 28604703 10.1038/nm.4357

[266] Schlechte J, Skalosky I, Geuking MB, McDonald B. Long-distance relationships—regulation of systemic host defense against infections by the gut microbiota. Mucosal Immunol. 2022;15(5):809‐818. 35732817 10.1038/s41385-022-00539-2

[267] Armitage SA, Genersch E, McMahon DP, Rafaluk-Mohr C, Rolff J. Tripartite interactions: how immunity, microbiota and pathogens interact and affect pathogen virulence evolution. Curr Opin Insect Sci. 2022;50:100871. 34999035 10.1016/j.cois.2021.12.011

[268] Hall MD, Bento G, Ebert D. The evolutionary consequences of stepwise infection processes. Trends Ecol Evol. 2017;32(8):612‐623. 28648806 10.1016/j.tree.2017.05.009

[269] Le Pendu J, Nyström K, Ruvoën-Clouet N. Host-pathogen co-evolution and glycan interactions. Curr Opin Virol. 2014;7:88‐94. 25000207 10.1016/j.coviro.2014.06.001

[270] Gerardo NM, Hoang KL, Stoy KS. Evolution of animal immunity in the light of beneficial symbioses. Philos Trans R Soc Lond B Biol Sci. 2020;375(1808):20190601. 32772666 10.1098/rstb.2019.0601 PMC7435162

[271] Shang W, Zhang S, Qian H, et al Gut microbiota and sepsis and sepsis-related death: a Mendelian randomization investigation. Front Immunol. 2024;15:1266230. 38361921 10.3389/fimmu.2024.1266230 PMC10867964

[272] Ippolito MM, Denny JE, Langelier C, Sears CL, Schmidt NW. Malaria and the microbiome: a systematic review. Clin Infect Dis. 2018;67(12):1831‐1839. 29701835 10.1093/cid/ciy374 PMC6260159

[273] Kullberg RFJ, Wiersinga WJ, Haak BW. Gut microbiota and sepsis: from pathogenesis to novel treatments. Curr Opin Gastroenterol. 2021;37(6):578‐585. 34419965 10.1097/MOG.0000000000000781

[274] Yang S, Guo J, Kong Z, et al Causal effects of gut microbiota on sepsis and sepsis-related death: insights from genome-wide Mendelian randomization, single-cell RNA, bulk RNA sequencing, and network pharmacology. J Transl Med. 2024;22(1):10. 38167131 10.1186/s12967-023-04835-8 PMC10763396

[275] Barlow B, Ponnaluri S, Barlow A, Roth W. Targeting the gut microbiome in the management of sepsis-associated encephalopathy. Front Neurol. 2022;13:999035. 36247756 10.3389/fneur.2022.999035 PMC9557965

[276] Caballero-Flores G, Pickard JM, Núñez G. Microbiota-mediated colonization resistance: mechanisms and regulation. Nat Rev Microbiol. 2023;21(6):347‐360. 36539611 10.1038/s41579-022-00833-7 PMC10249723

[277] Le Chatelier E, Nielsen T, Qin J, et al Richness of human gut microbiome correlates with metabolic markers. Nature. 2013;500(7464):541‐546. 23985870 10.1038/nature12506

[278] Roy P, Sugiyama K, Rao CD, Kusari J, Purdy M, Collisson E. Molecular epidemiology of two US orbiviruses: bluetongue virus and epizootic hemorrhagic disease virus. Prog Clin Biol Res. 1985;178:589‐595. 2989908

[279] Tilg H, Zmora N, Adolph TE, Elinav E. The intestinal microbiota fuelling metabolic inflammation. Nat Rev Immunol. 2020;20(1):40‐54. 31388093 10.1038/s41577-019-0198-4

[280] Thaiss CA, Itav S, Rothschild D, et al Persistent microbiome alterations modulate the rate of post-dieting weight regain. Nature. 2016;540(7634):544‐551. 27906159 10.1038/nature20796

[281] Rask-Madsen C, Kahn CR. Tissue-specific insulin signaling, metabolic syndrome, and cardiovascular disease. Arterioscler Thromb Vasc Biol. 2012;32(9):2052‐2059. 22895666 10.1161/ATVBAHA.111.241919 PMC3511859

[282] Caesar R, Tremaroli V, Kovatcheva-Datchary P, Cani PD, Bäckhed F. Crosstalk between gut microbiota and dietary lipids aggravates WAT inflammation through TLR signaling. Cell Metab. 2015;22(4):658‐668. 26321659 10.1016/j.cmet.2015.07.026 PMC4598654

[283] Xie L, Wang H, Wu D, et al CXCL13 promotes thermogenesis in mice via recruitment of M2 macrophage and inhibition of inflammation in brown adipose tissue. Front Immunol. 2023;14:1253766. 37936696 10.3389/fimmu.2023.1253766 PMC10627189

[284] Song X, Zhang H, Zhang Y, et al Gut microbial fatty acid isomerization modulates intraepithelial T cells. Nature. 2023;619(7971):837‐843. 37380774 10.1038/s41586-023-06265-4

[285] Di Filippo L, De Lorenzo R, Cinel E, et al Weight trajectories and abdominal adiposity in COVID-19 survivors with overweight/obesity. Int J Obes (Lond). 2021;45(9):1986‐1994. 34002039 10.1038/s41366-021-00861-y PMC8127478

[286] Hauner H, Blanken CPS, Holzapfel C. Long-lasting effects of the COVID-19 pandemic on lifestyle and body weight: results of representative cross-sectional surveys in adults in Germany. BMC Public Health. 2024;24(1):1199. 38684999 10.1186/s12889-024-18680-x PMC11059715

[287] Samuel M, Park RY, Eastwood SV, et al Trends in weight gain recorded in English primary care before and during the coronavirus-19 pandemic: an observational cohort study using the OpenSAFELY platform. PLoS Med. 2024;21(6):e1004398. 38913709 10.1371/journal.pmed.1004398 PMC11249215

[288] Shrestha DS, Manandhar S, Chalise BS, et al Symptoms 6 months following SARS-CoV-2 infection in Nepali women. PLoS One. 2024;19(3):e0299141. 38466665 10.1371/journal.pone.0299141 PMC10927087

[289] Lee SK, Lim Y, Jeong S, Han HW. COVID-19-related cardiovascular disease risk due to weight gain: a nationwide cohort study. Eur J Med Res. 2024;29(1):2. 38167158 10.1186/s40001-023-01569-7 PMC10762936

[290] Maiti AK . Bioinformatic analysis predicts the regulatory function of noncoding SNPs associated with long COVID-19 syndrome. Immunogenetics. 2024;76(5-6):279‐290. 39042286 10.1007/s00251-024-01348-6

[291] Fan J, Long QX, Ren JH, et al Genome-wide association study of SARS-CoV-2 infection in Chinese population. Eur J Clin Microbiol Infect Dis. 2022;41(9):1155‐1163. 35927536 10.1007/s10096-022-04478-5 PMC9362144

[292] Gómez-Carballa A, Pardo-Seco J, Pischedda S, et al Sex-biased expression of the TLR7 gene in severe COVID-19 patients: insights from transcriptomics and epigenomics. Environ Res. 2022;215(Pt 2):114288. 36152884 10.1016/j.envres.2022.114288 PMC9508271

[293] Ferreira LC, Gomes CEM, Rodrigues-Neto JF, Jeronimo SMB. Genome-wide association studies of COVID-19: connecting the dots. Infect Genet Evol. 2022;106:105379. 36280088 10.1016/j.meegid.2022.105379 PMC9584840

[294] Pairo-Castineira E, Rawlik K, Bretherick AD, et al GWAS and meta-analysis identifies 49 genetic variants underlying critical COVID-19. Nature. 2023;617(7962):764‐768. 37198478 10.1038/s41586-023-06034-3 PMC10208981

[295] COVID-19 Host Genetics Initiative . Mapping the human genetic architecture of COVID-19. Nature. 2021;600(7889):472‐477. 34237774 10.1038/s41586-021-03767-x PMC8674144

[296] Zhang D, Zhou Y, Ma Y, et al Gut microbiota dysbiosis correlates with long COVID-19 at one-year after discharge. J Korean Med Sci. 2023;38(15):e120. 37069814 10.3346/jkms.2023.38.e120 PMC10111044

[297] Álvarez-Santacruz C, Tyrkalska SD, Candel S. The microbiota in long COVID. Int J Mol Sci. 2024;25(2):1330. 38279329 10.3390/ijms25021330 PMC10816132

[298] Aranaz P, Ramos-Lopez O, Cuevas-Sierra A, Martinez JA, Milagro FI, Riezu-Boj JI. A predictive regression model of the obesity-related inflammatory status based on gut microbiota composition. Int J Obes (Lond). 2021;45(10):2261‐2268. 34267323 10.1038/s41366-021-00904-4

[299] Li Z, Xia Q, Feng J, et al The causal role of gut microbiota in susceptibility of long COVID: a Mendelian randomization study. Front Microbiol. 2024;15:1404673. 38873142 10.3389/fmicb.2024.1404673 PMC11169722

[300] Henneke L, Schlicht K, Andreani NA, et al A *dietary carbohydrate—gut Parasutterella—human fatty acid biosynthesis* metabolic axis in obesity and type 2 diabetes. Gut Microbes. 2022;14(1):2057778. 35435797 10.1080/19490976.2022.2057778 PMC9037427

